# Mapping the sex determination region in the *Salix* F_1_ hybrid common parent population confirms a ZW system in six diverse species

**DOI:** 10.1093/g3journal/jkac071

**Published:** 2022-03-25

**Authors:** Dustin G Wilkerson, Bircan Taskiran, Craig H Carlson, Lawrence B Smart

**Affiliations:** Horticulture Section, School of Integrative Plant Sciences, Cornell University, Cornell AgriTech, Geneva, NY 14456, USA

**Keywords:** QTL mapping, *Salix*, sex determination, linkage map, *Salix purpurea*, *Salix viminalis*, *Salix suchowensis*

## Abstract

Within the genus *Salix*, there are approximately 350 species native primarily to the northern hemisphere and adapted to a wide range of habitats. This diversity can be exploited to mine novel alleles conferring variation important for production as a bioenergy crop, but also to identify evolutionarily important genes, such as those involved in sex determination. To leverage this diversity, we created a mapping population by crossing 6 *Salix* species (*Salix viminalis*, *Salix suchowensis*, *Salix integra*, *Salix koriyanagi*, *Salix udensis*, and *Salix alberti*) to common male and female *Salix purpurea* parents. Each family was genotyped via genotyping-by-sequencing and assessed for kinship and population structure as well as the construction of 16 backcross linkage maps to be used as a genetic resource for breeding and selection. Analyses of population structure resolved both the parents and F_1_ progeny to their respective phylogenetic section and indicated that the *S. alberti* parent was misidentified and was most likely *S.suchowensis*. Sex determining regions were identified on *Salix* chromosome 15 in the female-informative maps for seven of the eight families indicating that these species share a common female heterogametic ZW sex system. The eighth family, *S. integra* × *S. purpurea*, was entirely female and had a truncated chromosome 15. Beyond sex determination, the *Salix* F_1_ hybrid common parent population (*Salix F_1_* HCP) introduced here will be useful in characterizing genetic factors underlying complex traits, aid in marker-assisted selection, and support genome assemblies for this promising bioenergy crop.

## Introduction 

The establishment of genomic resources is an important step in developing a fully realized breeding program, reinforced by modern tools for trait mapping, candidate gene identification, and marker-assisted selection. *Salix* and *Populus* (poplars) comprise the majority of species in the family Salicaceae, which consists of dioecious trees, shrubs, and subshrubs that are highly heterozygous. Shrub willow (*Salix* spp.) are grown in northern latitudes as a sustainable, high-yielding, carbon neutral, bioenergy crop that can grow on marginal land and provide multiple ecosystem services (Smart *et al.* 2005; Stoof *et al.* 2014; Clifton‐Brown *et al.* 2019; Fabio and Smart 2020). While shrub willow breeding in the United States has been active since the 1980’s, most of the nearly 350 species have yet to be tapped as a source of genetic diversity (Dickmann and Kuzovkina 2014; Stanton *et al.* 2014).

Genomic resources developed for poplar were used in early genomic studies in *Salix*, because their genomes are largely colinear (Hanley *et al.* 2006; Berlin *et al.* 2010). As sequencing technologies became increasingly more affordable, more *Salix* genome sequencing was completed and now there are several high-quality assemblies. Genomic resources for *Salix* are currently centered around a few key species. In Europe, *Salix viminalis* is an important bioenergy crop species with a recently published, high-quality genome assembly (Almeida *et al.* 2020). *Salix* *viminalis* has been used in several QTL mapping studies for resistance to willow leaf rust (*Melampsora larici-epitea*) (Rönnberg-Wästljung *et al.* 2008; Samils *et al.* 2011; Sulima *et al.* 2017), drought tolerance (Rönnberg-Wästljung *et al.* 2005), and growth and phenology (Hallingbäck *et al.* 2016; Hallingbäck *et al.* 2019). While in the United States, *Salix purpurea* is the model species for bioenergy willow breeding, genetics, and genomics. The US Department of Energy Joint Genome Institute has produced the highest quality annotated *Salix* reference genomes assembled in the genus on male and female *S. purpurea*, available on Phytozome (Zhou *et al.* 2018; Zhou *et al.* 2020; https://phytozome-next.jgi.doe.gov/info/SpurpureaFishCreek_v3_1; last accessed 29 March 2022; https://phytozome-next.jgi.doe.gov/info/Spurpurea_v5_1; last accessed 29 March 2022). Using joint linkage and association mapping approaches focused on *S. purpurea*, Carlson *et al.* (2019) identified numerous QTL for a wide range of morphological, physiological, insect and disease resistance and biomass composition traits. A naturalized species in North America, *S. purpurea* is a potential donor of broad adaptability traits for species susceptible to pests and diseases in the Northeast United States. There is value in studying the genomes of less-characterized *Salix* species for phylogenomic analysis and to discover diverse sources of alleles for introgression into elite yielding cultivars.

Here, we introduce the *Salix* F_1_ hybrid common parent population (*Salix* F_1_ HCP). The parents, described in Fabio *et al.* (2019) and Crowell *et al.* (2020), represent a diverse selection of species from *Salix* subgenus Vetrix (Dickmann and Kuzovkina 2014). Six *Salix* species (*S. viminalis*, *Salix* *suchowensis*, *Salix* *integra*, *Salix* *koriyanagi*, *Salix* *udensis*, and *Salix* *alberti*) were crossed to common male and female *S. purpurea* parents to form eight species hybrid families. Literature describing these species ranges from the high-quality reference genomes available for *S. purpurea* and *S. viminalis* to the scarcely studied *S. alberti* (Rosso *et al.* 2013). *Salix suchowensis* is native to China and has been used recently to generate a chromosome scale genome assembly (Wei *et al.* 2020). This species has been assessed for its response to drought stress (Jia *et al.* 2020) and was one of the first *Salix* species used to map the sex determination region (SDR) (Liu *et al.* 2013). *Salix udensis*, formally known as *S. sachalinensis*, has been described as a Japanese riparian willow species that acts as a natural nest cavity for fish owls (Niiyama 2008; Slaght *et al.* 2018) and is suggested to have sexually dimorphic characteristics (Ueno and Seiwa 2003; Ueno *et al.* 2006). Some genomic resources are available for the Korean *S. koriyanagi*, as its chloroplast genome have been sequenced (Kim *et al.* 2019; Park *et al.* 2019) while *S. integra* has been assessed for its phytoremediation potential (Cao *et al.* 2020; Yang *et al.* 2020b). Developed to interrogate the genetics of several understudied *Salix* species, the *Salix* F_1_ HCP is an important step in the development of genomic resources in *Salix*.

The Salicaceae represents an interesting family for the study of the evolution of dioecy and the mechanisms of sex determination. Genetic mapping of SDRs in the Salicaceae have revealed considerable intra- and inter-chromosomal variability and contrasting sex determination systems (Yang *et al.* 2021). Although Populus *alba* is female heterogametic (ZW) (Paolucci *et al.* 2010; Müller *et al.* 2020; Sabatti *et al.* 2020) and *Populus* *trichocarpa* is male heterogametic (XY) (Geraldes *et al.* 2015), both of their SDRs are located on chr19 while the SDR of *Populus* *euphratica* was identified on chr14 and also has an XY system (Yang *et al.* 2021). Conversely, the SDR of *S. purpurea* has been mapped to a large, pericentromeric region on *Salix* chr15, but has been found to maintain a few orthologous regions present within the *P. trichocarpa* chr19 SDR (Zhou *et al.* 2018). However, it is unclear whether dioecy evolved before or after their divergence (Hou *et al.* 2015). The sex systems of *Salix* also lack a consensus model. For instance, similar to *S. purpurea*, both *S. suchowensis* (Hou *et al.* 2015; Chen *et al.* 2016) and *S. viminalis* (Pucholt *et al.* 2015; Pucholt *et al.* 2017b; Hallingbäck *et al.* 2021) have female heterogametic (ZW) systems with SDRs located on *Salix* chr15, yet tree-form *S. nigra* has an XY system SDR on chr07 (Sanderson *et al.* 2021). Considering the variability in sex determination systems and SDR locations already discovered within *Salix*, elucidating the mechanisms of sex determination in more species could help to build a more cohesive understanding of SDR evolution. Further investigation could provide evidence to implicate SDR turnovers as a factor in the considerable species diversity within the genus.

To establish the *Salix* F_1_ HCP as a genetic resource, this study sought to: (1) describe the genetic relationships between the parent species, (2) develop female and male parent informative linkage maps for each family, and (3) define and compare their respective SDRs.

## Materials and methods

### Plant material

**Fig. 1.**
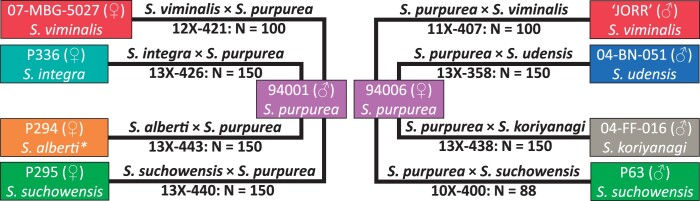
Crossing scheme of the *Salix* F_1_ HCP. There were four full-sib families with 94001 as the paternal parent and four full-sib families with 94006 as the maternal parent, both common parents are *S. purpurea*. Reciprocal crosses were made with male and female *S. viminalis* and *S. suchowensis* while *S. integra*, *S. alberti*, *S. udensis*, and *S. koriyanagi* were crossed only once. *Received as *S. alberti*.

Females of *S. viminalis*, *S. integra*, *S. alberti*, and *S. suchowensis* were crossed with male *S. purpurea* clone ID 94001, while males of *S. viminalis*, *S. udensis*, *S. koriyanagi*, and *S. suchowensis* were crossed with female *S. purpurea* clone ID 94006 creating eight F_1_ species hybrid families (Fig. 1). The common *S. purpurea* parents, 94006 and 94001, were chosen based upon their differential resistance to willow leaf rust (Crowell *et al.* 2020), adaptation to the Northeastern U.S., and availability of high-quality reference genomes (Zhou *et al.* 2020).

Crosses were made by forcing floral catkins of the parent genotypes from dormant shoots in a greenhouse. All crosses were made in isolation to prevent pollen contamination. When anthers began to dehisce, male catkins were excised and placed in falcon tubes for pollen extraction using toluene, as described in Kopp *et al.* (2002), then stored in 2 mL microcentrifuge tubes at ‒20°C until female catkins were receptive. Seedlings were established in a standard peat-based potting mix in a greenhouse, then transplanted to nursery beds near Cornell AgriTech (Geneva, NY, USA). Winter dormant cuttings from all parents and progeny were collected from 1-year-old stems and hand-planted in the field in a randomized complete block design with 3 plants per plot and 4 replicate blocks. Field trials were established with 1.83 m spacing between rows and 40.6 cm between plants within rows.

### DNA extraction and genotyping-by-sequencing

Shoot tips for DNA extraction were collected from plants in nursery beds and stored in desiccant. Dried shoot tips were ground to a fine powder with a Geno/Grinder (SPEX SamplePrep, Metuchen, NJ, USA) before genomic DNA extraction using the DNeasy Plant Mini Kit (QIAGEN Inc., Valencia, CA, USA). After checking the DNA quality using gel electrophoresis, DNA quantity was estimated using the Invitrogen Qubit dsDNA Broad Range Assay kit on a Qubit Fluorometer (ThermoFisher Scientific, Waltham, MA, USA). Genomic DNA was submitted to the University of Wisconsin Biotechnology Center (Madison, WI, USA) for 96-plex Genotyping-by-Sequencing (GBS) library preparation using the *ApeK*I restriction enzyme and sequenced (1 × 100 bp) on the Illumina HiSeq 2500 (Illumina, Inc., San Diego, CA, USA) platform.

### Variant discovery and imputation

Initial variant discovery and filtering were performed using the TASSEL GBS v2 Discovery Pipeline (Bradbury *et al.* 2007). Sequence reads were trimmed to 64 bp and aligned using BWA mem (Li and Durbin 2009) under default parameters to the *S. purpurea* v5.1 reference genome (Zhou *et al.* 2020). As this genome includes both chr15Z and 15W, chr15Z was excluded to reduce mapping errors within the pseudoautosomal regions surrounding the SDR in *S. purpurea*. This process was repeated once for all eight families together and then for each of the eight F_1_ families separately to identify population-wide and family-specific SNPs. By calling variants on all eight families and then on each family separately, variants were based on inter- and intra- familial genetic differences, respectively. The resulting VCF files contained 684,412 SNPs for the full analysis and ranged from 174,762 to 266,797 SNPs depending on the family. On the eight F_1_ datasets, SNPs with >70% missing data and minor allele frequency <0.01 and F_1_ individuals with >80% missing data or determined to be outliers based on principal component analysis were dropped. Missing genotype calls and low read depth are common in GBS (Elshire *et al.* 2011), therefore imputation was performed separately on each family to validate calls in haplotype blocks. Using LinkImputeR (Money *et al.* 2017), genotypes called with a read depth <5 were set to missing before filtering again for missingness >70%, resulting in 95,281 to 145,944 imputed SNPs with accuracies ranging from 84.3 to 93.1%.

### Population structure

SNPs for the population-wide analysis were filtered to retain markers and individuals with ≤20% missing data and SNPs with a minor allele frequency >0.01, which resulted in 55,398 SNPs. Multiple sequence runs of each of the parents were included as technical replicates for greater confidence in GBS calls. Analysis of principal components, once with just the parents and then again with the full population, was performed using default parameters in Tassel 5 and visualized in R (R Core Team 2020). Using only the parents, Tassel 5 was also used to generate a distance matrix and subsequent unrooted neighbor-joining tree for phylogenetic analysis. The parental runs and 10 randomly selected F_1_ progeny were analyzed using fastSTRUCTURE (Raj *et al.* 2014). Only a subset of the F_1_ were used in this analysis to manage file size and computation requirements. Multiple analyses of “structure.py” were completed (*K* = 3–10). Using “chooseK.py,” *K* = 6 represented the model complexity that maximized the marginal likelihood and best explained the data structure, suggesting six separate populations.

### Linkage map construction and analysis

Unless otherwise indicated, linkage map construction and analysis were performed using custom R code, available on Github (link in Data Availability). Multiple runs of each parent were used to form consensus genotypes for each SNP. In the absence of a clear consensus, the genotype was set to missing. If both parents were set to missing, the SNP was removed from the analysis. If only one parent was set to missing, its genotype was inferred based on the genotype of the known parent and the segregation of the F_1_. These parental consensus genotypes were used to identify the female informative (AB × AA) and male informative (AA × AB) markers used to generate backcross linkage maps for each parent using a combination of R/qtl (Broman *et al.* 2003) and ASMap (Taylor and Butler 2017). Co-located markers and those exhibiting extreme segregation distortion were removed using ASMap function “pullCross.” Linkage groups were then created using “mstmap” with default parameters except for objective.fun = “ML” and bychr = FALSE. The *p*-value for determining linkage groups varied between families, ranging from 1e-6 to 1e-12, depending on the demarcation of the 19 expected linkage groups. A custom R function was then used to perform simple error correction to reduce the number of double crossovers and deflate map distances before reforming linkage groups. Briefly, this function relies on the marker order determined after formation of linkage groups, identifying double crossovers of ≤2 SNPs, and correcting them. Map quality was checked using two strategies. The “heatMap” function in ASMap, which plots LOD linkage between markers on the upper triangle and estimated recombination frequency on the bottom, reveals markers that are problematic or out of phase. Then by comparing the physical position (Mb) based on alignment to the *S. purpurea* reference genome and the genetic distance (cM) within each linkage group shows issues with marker order or potential chromosomal rearrangements.

To delimit the SDR in each family, the sex of all F_1_ individuals was recorded by inspecting flowering catkins during two growing seasons, on plants with 2 and 3 years of post-coppice regrowth. Sex ratio bias was tested using a chi-square test for a 1:1 sex ratio. Using functions in R/qtl, genotype probabilities were calculated using “calc.genoprob” (step = 0, error.prob = 0.01, map.function = “kosambi,” stepwidth = “fixed”). Next, QTL mapping for individual sex was performed using “scanone”: model = “binary,” method = “em.” Genome-wide significance thresholds were determined based on the results of “scanone.perm” (n.perm = 1000). QTL positions were refined using “refineqtl” (method = “hk,” model = “binary”), then the 1.5 LOD support intervals were calculated using “lodint” with default parameters.

## Results

### Population structure

**Fig. 2.**
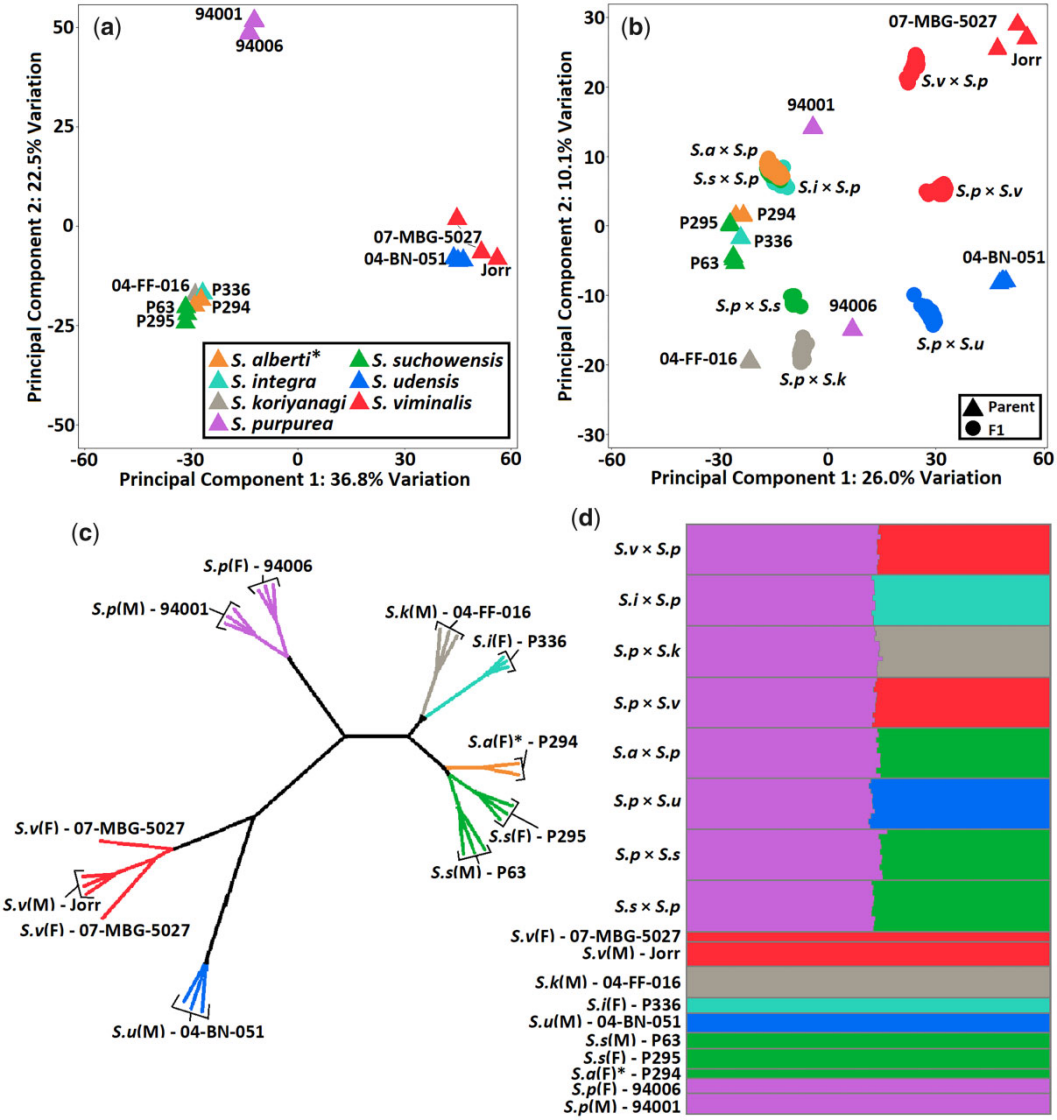
Results of PC, phylogenetic, and fastSTRUCTURE analysis of the *Salix* F_1_ HCP. Multiple technical replicates of the parents were included. a) PCA of the parents; b) PCA of the F_1_ and the parents; c) Unrooted neighbor-joining tree of the parents; d) Distruct plot using fastSTRUCTURE results. The species legend in panel (a) applies to panels (b) and (c) while colors in panel (d) are based on fastSRUCTURE results, affecting only *S. alberti*. *Received as *S. alberti*.

A combination of PCA, phylogenetic analysis, and fastSTRUCTURE were used to describe the population structure of the *Salix* F_1_ HCP. For clarity when referring to specific individuals, the plant species is abbreviated to its first letter and includes its sex, M or F, in parenthesis. A PCA of multiple sequence runs of the parents revealed three distinct clusters, formed by two PCs accounting for 36.8% and 22.5% of the total genetic variation (Fig. 2a). The two common parents, *S. p*(F) 94006 and *S. p*(M) 94001, formed a single group and were separated from the other parents by PC2. The *S. viminalis* parents, ‘Jorr’ (M) and 07-MBG-5027 (F), formed a cluster with *S. u*(M) 04-BN-051, which was differentiated from the remaining species by PC1. Including the F_1_ progeny from each family into the analysis, each PC accounted for 26% and 10.1% of the total variation (Fig. 2b). PC2 split *S. udensis* from the two *S. viminalis* parents and separated *S. koriyanagi* from *S. suchowensis*, *S. integra*, and *S. alberti*. As expected, the F_1_ individuals were intermediate between the common parent and family-specific species. The F_1_ progeny derived from the female parents *S. s*(F) P295, *S. a*(F) P294, and *S. i*(F) P336 (all crossed with *S. p*(M) 94001), co-localized.

Kinship analysis grouped the two *S. viminalis* parents with *S. udensis*, the two *S. purpurea* parents together, and *S. koriyanagi* with *S. integra* near *S. suchowensis* and *S. alberti* (Fig. 2c). These results closely mirrored the separation attributable to PC1 (Fig. 2a). In both PC and phylogenetic analyses, *S.a*(F) P294 grouped closely with both *S. suchowensis* parents. Admixture analysis included the parents and a subset of the F_1_ individuals of each family. The eight groups of F_1_ individuals were comprised of roughly half the genetic background of *S. purpurea* and half the other species parent, as expected (Fig. 2d). *Salix viminalis*, *S. integra*, *S. koriyanagi*, *S. udensis* and *S. purpurea* formed distinct populations, while *S. a*(F) P294 grouped together with the two *S. suchowensis* parents.

### Linkage map construction and analysis

**Fig. 3.**
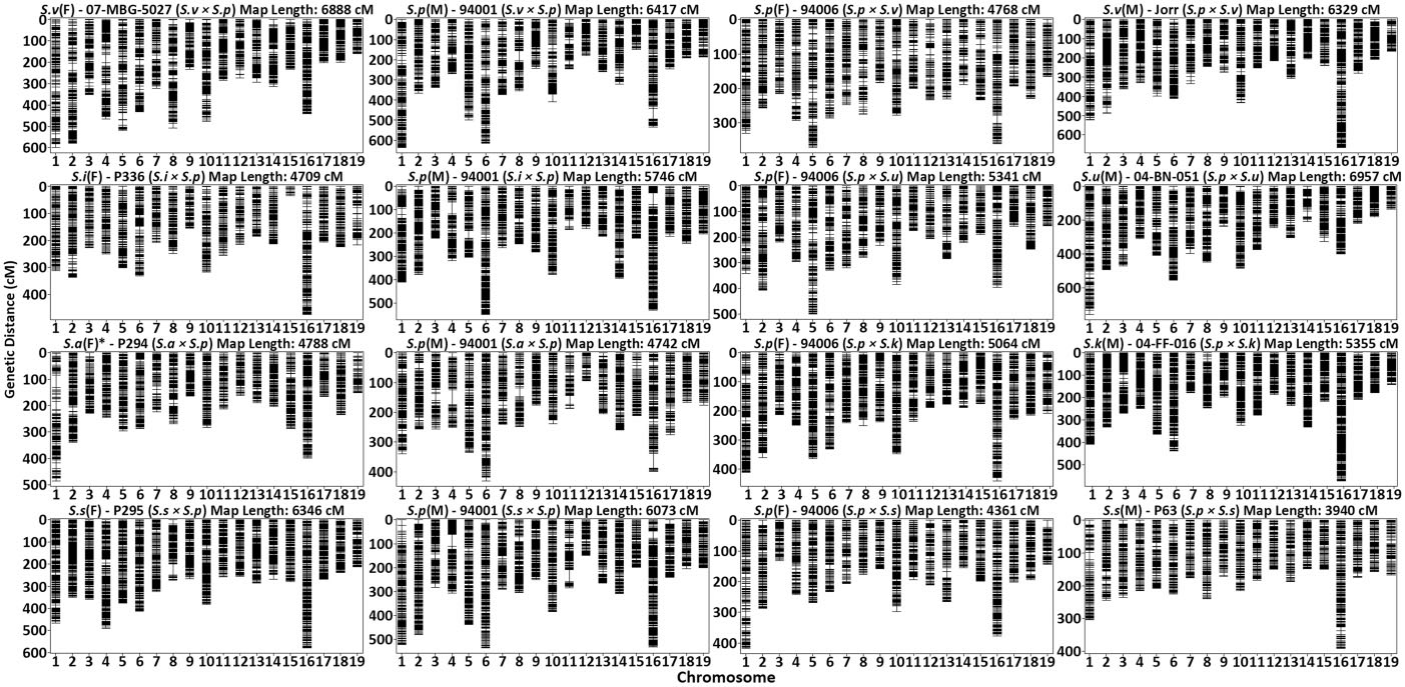
Linkage maps for each of the parents within the *Salix* F_1_ HCP. Female maps (first and third columns) were constructed using female informative markers (AB × AA), while male maps (second and forth columns) were constructed using male informative markers (AA × AB). Each map title can be read as: abbreviated species (sex)—parent name (family pedigree). Total and chromosome-specific summary statistics for each map are provided in Supplementary Table 1. *Received as *S. alberti.*

**Fig. 4.**
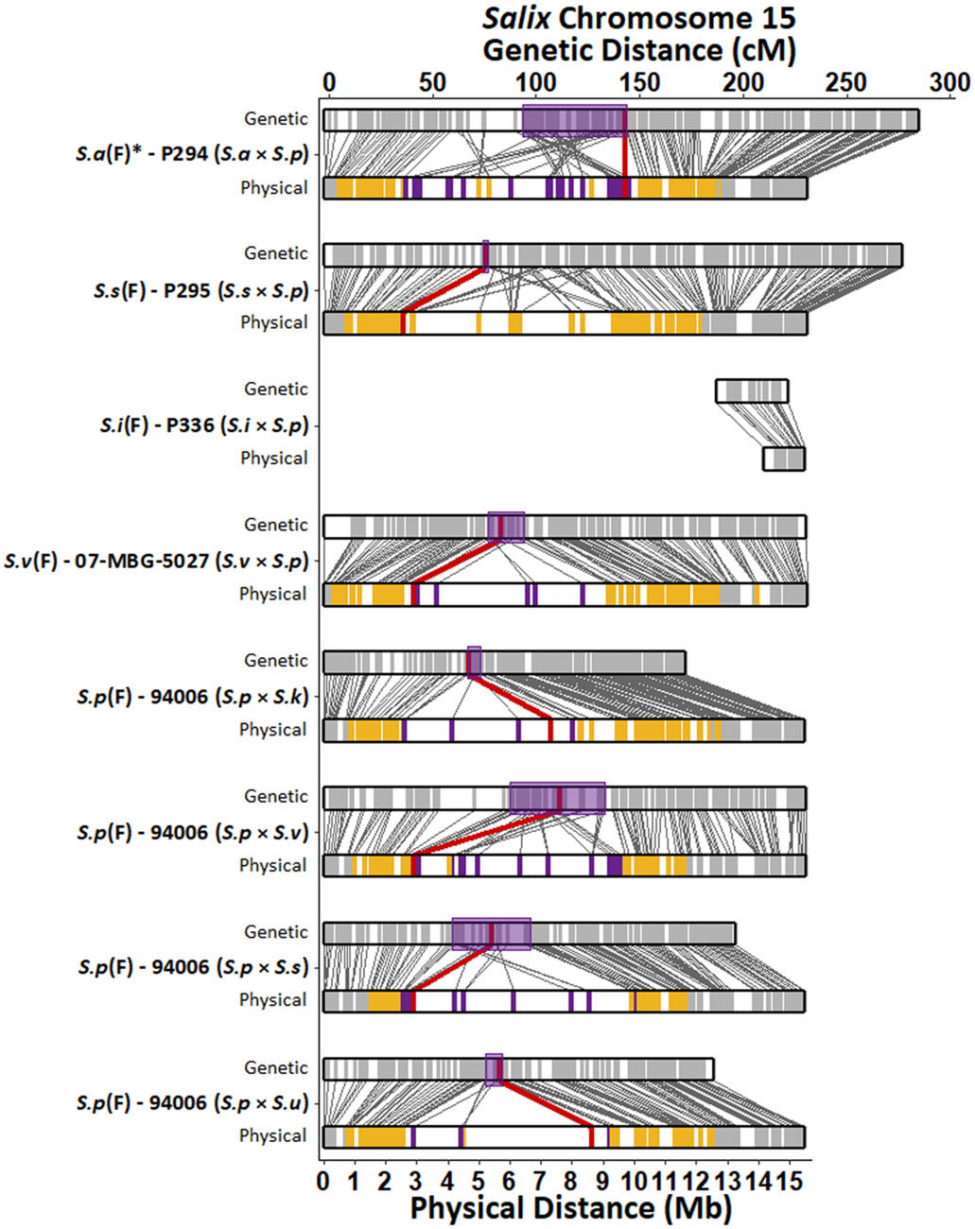
Genetic (cM) and physical (Mb) distances of the *Salix* F_1_ HCP maternal parent’s chromosome 15 with mapped QTL for sex. Each parent has a set of maps, genetic on the top and physical on the bottom with lines connecting them indicating a marker's relative position in both maps. On the genetic maps, each marker is represented by a gray vertical line while the 1.5 LOD support interval of the QTL is shown with a purple box. On the physical map, gray vertical lines are markers not associated with the QTL, yellow lines are markers whose LOD score was above the permuted significance threshold, purple lines are markers within the 1.5 LOD support interval, and red lines indicate the position of the peak marker. The cM distance of *S.i*(F) P336's linkage map was artificially increased for figure clarity. *Received as *S. alberti.*

Since there is considerable divergence between parents and pedigrees, variant discovery and marker filtration were performed for each family separately. Using consensus genotypes derived from multiple sequencing runs of the parents, markers were split into female (AB × AA) and male (AA × AB) informative backcross markers for linkage map construction, which resulted in 16 linkage maps (Fig. 3). Each linkage map consisted of 19 linkage groups with total map lengths ranging from 3939.9–6957.3 cM containing between 2035 and 3852 total markers (Supplementary Table 1). Recombination frequency and genetic to physical distance plots generated for each linkage map revealed that marker order and phase within each linkage group were reasonably linear (Supplementary Fig. 1). Sex phenotypes were used for QTL mapping of the SDR. Six of the eight families displayed significant sex ratio bias toward females based on a simple chi-square test (*P* < 0.05), with female to male ratios ranging from 1.4 to 1.6 (Table 1). Neither of the *S. viminalis* families displayed sex ratio bias, while the *S. integra* × *S. purpurea* family was entirely female. Single QTL for sex were identified on chr15 within seven of the eight maternal maps, excluding *S. i*(F) P336 of the *S. integra* × *S. purpurea* family. Each QTL explained between 60.7% and 74.1% of the total phenotypic variation (Table 2). LOD scores for the female and male linkage maps are in Supplementary Tables 2 and 3, respectively. Genome-wide LOD significance thresholds ranged from 3.39 to 3.69, depending on the map. On the four maternal *S. p*(F) 94006 maps, the QTL as determined by the permuted significance thresholds accounted for 10.3–12.03 Mb of the roughly 15.5 Mb chr15 due to suppressed recombination (Fig. 4). QTL refinement and 1.5 LOD support intervals narrowed this down to span 1.17–7.31 Mb with peak markers located at 2.91, 2.92, 7.33, and 8.65 Mb when *S. s*(M) P63, *S. v(M)* Jorr, *S. k*(M) 04-FF-016, and *S. u*(M) 04-BN-051 were the paternal parent, respectively (Table 2; Fig. 4). Of the three remaining maternal maps, QTL determined by LOD significance accounted for 12.3, 11.45, and 13.55 Mb of chr15 on the *S. a*(F) P294, *S. s*(F) P295, and *S. v*(F) 07-MBG-5027 maps, respectively, reflecting suppressed recombination. Upon refinement, these ranges were narrowed to account for 1.59, 6.91, and 6.41 Mb of the chromosome, with peaks centered at 9.73, 2.57, and 2.92 Mb.

**Table 1.** Sex phenotype statistics for the eight families in the *Salix* F_1_ HCP.

Family	Maternal species	Paternal species	*N*	Females	Males	F:M Ratio	*P*-value^a^
*S.p *×* S.u*	*S. purpurea*	*S. udensis*	150	91	59	1.6:1	0.01*
*S.p *×* S.s*	*S. purpurea*	*S. suchowensis*	88	53	33	1.6:1	0.03*
*S.p *×* S.v*	*S. purpurea*	*S. viminalis*	100	60	40	1.5:1	0.05^NS^
*S.p *×* S.k*	*S. purpurea*	*S. koriyanagi*	150	93	57	1.6:1	<0.001*
*S.v *×* S.p*	*S. viminalis*	*S. purpurea*	100	53	47	1.1:1	0.55^NS^
*S.i *×* S.p*	*S. integra*	*S. purpurea*	150	150	0	1:0	<0.001*
*S.s *×* S.p*	*S. suchowensis*	*S. purpurea*	150	91	58	1.6:1	0.01*
*S.a *×* S.p*	*S. alberti* ^b^	*S. purpurea*	150	87	62	1.4:1	0.04*

* indicates significance in chi-square test for sex ratio bias (*P* < 0.05).

NS Not significant.

b Received as *S. alberti*.

**Table 2.** Sex QTL associated with the SDR within the maternal linkage maps.

Family	Maternal Species	Chr	Peak (cM)	Peak LOD	Peak (Mb)	Low-LSI (Mb)	High-LSI (Mb)	VE (%)
*S.p *×* S.u*	*S. purpurea*	15	85.3	29.3	8.65	4.51	9.22	73.7
*S.p *×* S.s*	*S. purpurea*	15	81.1	16.1	2.91	2.61	9.92	61.8
*S.p *×* S.v*	*S. purpurea*	15	114.2	18.6	2.92	2.88	4.05	60.7
*S.p *×* S.k*	*S. purpurea*	15	70.1	35.1	7.33	2.63	8.29	71.1
*S.v *×* S.p*	*S. viminalis*	15	86.0	21.1	2.92	2.92	9.36	66.0
*S.s *×* S.p*	*S. suchowensis*	15	78.4	26.7	2.57	2.44	9.35	74.1
*S.a *×* S.p*	*S. alberti^a^*	15	145.8	35.6	9.73	8.63	10.22	70.3

LSI, 1.5 LOD support interval; VE, variation explained; cM, centiMorgan position within the linkage map; Mb, physical position based on alignment to *S. purpurea* reference genome.

a Received as *S. alberti*.

## Discussion

*Salix* is a very diverse genus, consisting of more than 350 species. That diversity extends to variation between species even in traits as evolutionarily important as sex determination (Yang *et al.* 2021). By generating mapping populations that include characterized species crossed with those less studied, we will increase the number of *Salix* species available for trait introgression in breeding programs. We developed the *Salix* F_1_ HCP as a resource to characterize the interactions between alleles from different, but related species, and to map variation in important traits. Using GBS, we analyzed the population structure among the eight families, generated linkage maps of each of the parents using backcross markers and mapped the SDR in seven of the eight families using phenotypes collected from repeated field surveys. PCA predominately resolved the population by section with the F_1_ clustering between the parents as expected. In both phylogenetic and fastSTRUCTURE analyses, *S. a*(F) P294 was found to be very closely related to the *S. suchowensis* parents. Given these results and the limited publicly available information about *S. alberti*, P294 is likely *S. suchowensis* and will be described as such.

Among the 16 linkage maps produced, all QTL for sex were detected on chr15 in seven of the eight families and only in the maternal maps. In a recent study mapping sex in *S. triandra* using backcross markers, Li *et al.* (2020) were also only able to detect QTL within the maternal map, which is indicative of a ZW sex determination system on chr15. While this had been known for *S. purpurea* (Zhou *et al.* 2018), *S. viminalis* (Pucholt *et al.* 2017b; Hallingbäck *et al.* 2021), and *S. suchowensis* (Chen *et al.* 2016), this is the first study to report that both *S. koriyanagi* and *S. udensis* have ZW SDR on chr15.

Although the location of the SDR has also not been reported in *S. integra*, the *S. integra* × *S. purpurea* family was entirely female and therefore was excluded in the linkage analysis. The chr15 map from *S. i*(F) P336 is considerably smaller than the other families, aligning to the 14.2–15.5 Mb region of the *S. purpurea* reference genome, while maps from the other families had near complete coverage. The region of *S.* i(F) P336’s chr15 that did have segregating markers was outside the SDR intervals of the other female maps and is likely in the pseudoautosomal region of the sex chromosome.

Six of the eight families were female biased—a feature prevalent in *Salix*. Of those studied here, *S. purpurea* (Gouker *et al.* 2021), *S. viminalis* (Alström-Rapaport *et al.* 1997; Pucholt *et al.* 2017a), *S. suchowensis* (Yang *et al.* 2020a), *S. udensis* (Ueno and Seiwa 2003), and *S. integra* (Tozawa *et al.* 2009) have documented cases of sex ratio bias, yet this is the first time it has been reported in *S. koriyanagi*. The genetic basis of sex ratio bias could be a result of secondary sex dimorphisms, such as higher mortality rates in males, increased herbivory and pathogen resistance in females, or the presence of a sex distorter locus (Pucholt *et al.* 2017a).

Mapping of the SDR in this study resulted in marker associations that covered a majority of chr15 prior to refinement. Of the four families with *S. p*(F) 94006 as the common female parent, refined QTL shared similar ranges of the SDR described by Zhou *et al.* (2020) (6.8 Mb in length, starting at 2.3 Mb) with the exception of the *S. purpurea* × *S. viminalis* family, which was 1.17 Mb in length. Carlson *et al.* (2019) mapped the SDR to 4.5–11.4 Mb on chr15 using a *S. purpurea* F_2_ population while this study localized the SDR to the same pericentromeric region of chr15 in *S. purpurea* using F_1_ hybrid families.

The most recent delimitation of the SDR in *S. viminalis* spanned roughly 3.4 Mb (approx. 2.3–5.7 Mb) of chr15 (Almeida *et al.* 2020) and through Hallingbäck *et al.* (2021), is most likely controlled by a single locus. Our mapping of the SDR overlaps this region by 2.75 Mb, including the position of our peak marker, even though we aligned markers to a different reference genome. Almeida *et al.* (2020) aligned the SDR to the chr15 of both *S. purpurea* v1.0 and *S. viminalis* reference genomes and found overall synteny between species, yet with several structural rearrangements. This contrasts with what is seen in our results, where the chr15 of *S.v*(F) 07-MBG-5027 showed only minor rearrangements, likely due to alignment to the high-quality *S. purpurea* v5.1 reference genome.

Based on annotations from the *P. trichocarpa* genome, differential gene expression analysis in *S. suchowensis* between male and female plants led to predictions that the SDR was originally on chr14 (Liu *et al.* 2013). However, later work repositioned the SDR to the centromeric region of chr15 when based on *Salix* alignment (Chen *et al.* 2016). Our study defined the physical distance of the SDR at 2.44–9.35 Mb in *S.s*(F) P295 and 8.63–10.22 Mb in *S.s*(F) P294 with an overlap of 0.72 Mb. These two linkage maps show the greatest amount of rearrangement on chr15 when aligned to the *S. purpurea* reference. It is fair to conclude that alignment to a future *S. suchowensis* reference genome would aid in improving the mapping resolution in this region.

Suppressed recombination is a hallmark of chromosomes containing an SDR. Comparing the map of chr15 from each family, the SDR extends across a region of sparce marker density, approximately 3–9 Mb and is flanked by regions with greater marker density. As described above, this centromeric region with suppressed recombination is often associated with the SDR in *Salix*. In all seven families with mapped SDR, the peak marker was located within this region although its position varied. In the two *S. viminalis* families and two of the three *S. suchowensis* families, the peak marker was located within a 0.38 Mb region (2.57–2.95 Mb), while the remaining three families were less consistent and located proximal to the centromere. The generation of additional reference genomes for use in mapping will add context to these results and further refine the structure of the SDR among variable species.

This study described the population structure among the eight families within the *Salix* F_1_ HCP, constructed linkage maps for each parent, and mapped the SDR to the maternal chr15 in seven of the eight families. The introduction of the *Salix* F_1_ HCP provides the opportunity to map QTL for phenotypic traits beyond sex determination, while the linkage maps could be used to anchor and scaffold contigs in the generation of new reference genomes for each of the parents. While all species have a ZW sex determination system with an SDR that maps to chr15, these genetic resources provide a foundation for further characterization of the mechanism of sex determination and mapping of other key traits in these related species.

## Data availability

The GBS data used in the population structure analysis and the eight family-specific files prior to linkage map construction are available in hapmap format through figshare at https://figshare.com at “GBS Data from Wilkerson *et al.*” (https://figshare.com/articles/dataset/GBS_Data_from_Wilkerson_et_al_2021/16926043; last accessed 29 March 2022). R code used to format and create the linkage maps is available on the Willowpedia Github site located at https://github.com/Willowpedia/Wilkerson_etal_SalixLinkageMaps; last accessed 29 March 2022. Supplementary Table 1 contains statistics on each of the 16 linkage maps created in this study, including marker count and cM length for each linkage group and the total markers and cM length for each map. Supplementary Fig. 1 is a PDF slide show that, one map per slide, shows the marker cM by physical position for each linkage group and a heatmap visualizing recombination frequency and linkage. Supplementary Tables 2 and 3 contain the cM distance and sex QTL LOD scores of markers within each female and male map, respectively.

Supplemental material is available at *G3* online.

## Supplementary Material

jkac071_Supplementary_Data

## References

[jkac071-B1] Almeida P , Proux-Wera E, Churcher A, Soler L, Dainat J, Pucholt P, Nordlund J, Martin T, Rönnberg-Wästljung A-C, Nystedt B, et al Genome assembly of the basket willow, *Salix viminalis*, reveals earliest stages of sex chromosome expansion. BMC Biol. 2020;18(1):78. 32605573 10.1186/s12915-020-00808-1 PMC7329446

[jkac071-B2] Alström-Rapaport C , Lascoux M, Gullberg U. Sex determination and sex ratio in the dioecious shrub *Salix viminalis* L. Theor Appl Genet. 1997;94(3–4):493–497.

[jkac071-B3] Berlin S , Lagercrantz U, von Arnold S, Ost T, Ronnberg-Wastljung AC. High-density linkage mapping and evolution of paralogs and orthologs in *Salix* and *Populus*. BMC Genomics. 2010;11:129. 20178595 10.1186/1471-2164-11-129 PMC2834636

[jkac071-B4] Bradbury PJ , Zhang Z, Kroon DE, Casstevens TM, Ramdoss Y, Buckler ES. TASSEL: software for association mapping of complex traits in diverse samples. Bioinformatics. 2007;23(19):2633–2635. 17586829 10.1093/bioinformatics/btm308

[jkac071-B5] Broman KW , Wu H, Sen Ś, Churchill GA. R/qtl: QTL mapping in experimental crosses. Bioinformatics. 2003;19(7):889–890. 12724300 10.1093/bioinformatics/btg112

[jkac071-B6] Cao Y , Ma C, Chen H, Zhang J, White JC, Chen G, Xing B. Xylem-based long-distance transport and phloem remobilization of copper in *Salix integra* Thunb. J Hazard Mater. 2020;392:122428. 32208308 10.1016/j.jhazmat.2020.122428

[jkac071-B7] Carlson CH , Gouker FE, Crowell CR, Evans L, DiFazio SP, Smart CD, Smart LB. Joint linkage and association mapping of complex traits in shrub willow (*Salix purpurea* L.). Ann Bot. 2019;124(4):701–716. 31008500 10.1093/aob/mcz047 PMC6821232

[jkac071-B8] Chen Y , Wang T, Fang L, Li X, Yin T. Confirmation of single-locus sex determination and female heterogamety in willow based on linkage analysis. PLoS One. 2016;11(2):e0147671. 26828940 10.1371/journal.pone.0147671 PMC4734660

[jkac071-B9] Clifton-Brown J , Harfouche A, Casler MD, Dylan Jones H, Macalpine WJ, Murphy-Bokern D, Smart LB, Adler A, Ashman C, Awty-Carroll D, et al Breeding progress and preparedness for mass‐scale deployment of perennial lignocellulosic biomass crops switchgrass, miscanthus, willow and poplar. Glob Change Biol Bioenergy. 2019;11(1):118–151. 30854028 10.1111/gcbb.12566 PMC6392185

[jkac071-B10] Crowell CR , Bekauri MM, Cala AR, McMullen P, Smart LB, Smart CD. Differential susceptibility of diverse *Salix* spp. to *Melampsora americana* and *Melampsora paradoxa*. Plant Dis. 2020;104(11):2949–2957. 32902356 10.1094/PDIS-04-20-0718-RE

[jkac071-B11] Dickmann DI , Kuzovkina J. Poplars and Willows of the World, with Emphasis on Silviculturally Important Species. Poplars and Willows: trees for Society and the Environment. Rome (Italy): FAO; 2014.

[jkac071-B12] Elshire RJ , Glaubitz JC, Sun Q, Poland JA, Kawamoto K, Buckler ES, Mitchell SE. A robust, simple genotyping-by-sequencing (GBS) approach for high diversity species. PLoS One. 2011;6(5):e19379. 21573248 10.1371/journal.pone.0019379 PMC3087801

[jkac071-B13] Fabio ES , Leary CJ, Smart LB. Tolerance of novel inter-specific shrub willow hybrids to water stress. Trees. 2019;33(4):1015–1026.

[jkac071-B14] Fabio ES , Smart LB. Genetic and environmental influences on first rotation shrub willow (*Salix* spp.) bark and wood elemental composition. Bioenergy Res. 2020;13(3):797–809.

[jkac071-B15] Geraldes A , Hefer CA, Capron A, Kolosova N, Martinez-Nuñez F, Soolanayakanahally RY, Stanton B, Guy RD, Mansfield SD, Douglas CJ, et al Recent Y chromosome divergence despite ancient origin of dioecy in poplars (*Populus*). Mol Ecol. 2015;24(13):3243–3256. 25728270 10.1111/mec.13126

[jkac071-B16] Gouker FE , Carlson CH, Zou J, Evans L, Crowell CR, Smart CD, DiFazio SP, Smart LB. Sexual dimorphism in the dioecious willow Salix purpurea. Am J Bot. 2021;108(8):1374–1387. 34406658 10.1002/ajb2.1704

[jkac071-B17] Hallingbäck HR , Berlin S, Nordh N-E, Weih M, Rönnberg-Wästljung A-C. Genome wide associations of growth, phenology, and plasticity traits in willow [*Salix viminalis* (L.)]. Front Plant Sci. 2019;10:753. 31249579 10.3389/fpls.2019.00753 PMC6582754

[jkac071-B18] Hallingbäck HR , Fogelqvist J, Powers SJ, Turrion-Gomez J, Rossiter R, et al Association mapping in *Salix viminalis* L. (Salicaceae)—identification of candidate genes associated with growth and phenology. GCB Bioenergy. 2016;8(3):670–685. 27547245 10.1111/gcbb.12280 PMC4973673

[jkac071-B19] Hallingbäck HR , Pucholt P, Ingvarsson PK, Rönnberg-Wästljung A-C, Berlin S. Genome-wide association mapping uncovers sex-associated copy number variation markers and female hemizygous regions on the W chromosome in *Salix viminalis*. BMC Genomics. 2021;22(1):710. 34600471 10.1186/s12864-021-08021-2 PMC8487499

[jkac071-B20] Hanley S , Mallott M, Karp A. Alignment of a *Salix* linkage map to the *Populus* genomic sequence reveals macrosynteny between willow and poplar genomes. Tree Genet Genomes. 2006;3(1):35–48.

[jkac071-B21] Hou J , Ye N, Zhang D, Chen Y, Fang L, Dai X, Yin T. Different autosomes evolved into sex chromosomes in the sister genera of *Salix* and *Populus*. Sci Rep. 2015;5(1):9076. 25766834 10.1038/srep09076 PMC4357872

[jkac071-B22] Jia H , Wang L, Li J, Sun P, Lu M, et al Physiological and metabolic responses of *Salix sinopurpurea* and *Salix suchowensis* to drought stress. Trees. 2020;34(2):563–577.

[jkac071-B23] Kim J , Kim Y, Park J. Complete chloroplast genome sequence of the *Salix koriyanagi* Kimura ex Goerz (Salicaceae). Mitochondrial DNA B. 2019;4(1):549–550.

[jkac071-B24] Kopp R , Maynard C, Rocha De Niella P, Smart L, Abrahamson L. Collection and storage of pollen from *Salix* using organic solvents. Am J Bot. 2002;89(2):248–252. 21669733 10.3732/ajb.89.2.248

[jkac071-B25] Li H , Durbin R. Fast and accurate short read alignment with Burrows-Wheeler transform. Bioinformatics. 2009;25(14):1754–1760. 19451168 10.1093/bioinformatics/btp324 PMC2705234

[jkac071-B26] Li W , Wu H, Li X, Chen Y, Yin T. Fine mapping of the sex locus in *Salix triandra* confirms a consistent sex determination mechanism in genus *Salix*. Hortic Res. 2020;7(1):64. 32377355 10.1038/s41438-020-0289-1 PMC7193568

[jkac071-B27] Liu J , Yin T, Ye N, Chen Y, Yin T, Liu M, Hassani D. Transcriptome analysis of the differentially expressed genes in the male and female shrub willows (*Salix suchowensis*). PLoS One. 2013;8(4):e60181. 23560075 10.1371/journal.pone.0060181 PMC3613397

[jkac071-B28] Money D , Migicovsky Z, Gardner K, Myles S. LinkImputeR: user-guided genotype calling and imputation for non-model organisms. BMC Genomics. 2017;18(1):523. 28693460 10.1186/s12864-017-3873-5 PMC5504746

[jkac071-B29] Müller NA , Kersten B, Leite Montalvão AP, Mähler N, Bernhardsson C, Bräutigam K, Carracedo Lorenzo Z, Hoenicka H, Kumar V, Mader M, et al A single gene underlies the dynamic evolution of poplar sex determination. Nat Plants. 2020;6(6):630–637. 32483326 10.1038/s41477-020-0672-9

[jkac071-B30] Niiyama K. Coexistence of Salix species in a seasonally flooded habitat. In: Sakio H, Tamura T, editors. Ecology of Riparian Forests in Japan. New York (USA): Springer; 2008. p. 165–174.

[jkac071-B31] Paolucci I , Gaudet M, Jorge V, Beritognolo I, Terzoli S, et al Genetic linkage maps of *Populus alba* L. and comparative mapping analysis of sex determination across *Populus* species. Tree Genet Genomes. 2010;6(6):863–875.

[jkac071-B32] Park J , Kim Y, Xi H. The complete chloroplast genome sequence of male individual of Korean endemic willow, *Salix koriyanagi* Kimura ex Goerz (Salicaceae). Mitochondrial DNA. 2019;4(1):1619–1621.

[jkac071-B33] Pucholt P , Hallingbäck HR, Berlin S. Allelic incompatibility can explain female biased sex ratios in dioecious plants. BMC Genomics. 2017a;18(1):251. 28335728 10.1186/s12864-017-3634-5 PMC5364565

[jkac071-B34] Pucholt P , Rönnberg-Wästljung AC, Berlin S. Single locus sex determination and female heterogamety in the basket willow (*Salix viminalis* L.). Heredity (Edinb). 2015;114(6):575–583. 25649501 10.1038/hdy.2014.125 PMC4434249

[jkac071-B35] Pucholt P , Wright AE, Conze LL, Mank JE, Berlin S. Recent sex chromosome divergence despite ancient dioecy in the willow *Salix viminalis*. Mol Biol Evol. 2017b;34(8):1991–2001. 28453634 10.1093/molbev/msx144 PMC5850815

[jkac071-B36] R Core Team. R: A Language and Environment for Statistical Computing. Vienna (Austria): R Foundation for Statistical Computing; 2020.

[jkac071-B37] Raj A , Stephens M, Pritchard JK. fastSTRUCTURE: variational inference of population structure in large SNP data sets. Genetics. 2014;197(2):573–589. 24700103 10.1534/genetics.114.164350 PMC4063916

[jkac071-B38] Rönnberg-Wästljung AC , Glynn C, Weih M. QTL analyses of drought tolerance and growth for a *Salix dasyclados* x *Salix viminalis* hybrid in contrasting water regimes. Theor Appl Genet. 2005;110(3):537–549. 15619077 10.1007/s00122-004-1866-7

[jkac071-B39] Rönnberg-Wästljung AC , Samils B, Tsarouhas V, Gullberg U. Resistance to *Melampsora larici-epitea* leaf rust in *Salix*: analyses of quantitative trait loci. J Appl Genet. 2008;49(4):321–331. 19029679 10.1007/BF03195630

[jkac071-B40] Rosso L , Facciotto G, Bergante S, Vietto L, Nervo G. Selection and testing of *Populus alba* and *Salix* spp. as bioenergy feedstock: preliminary results. Appl Energy. 2013;102:87–92.

[jkac071-B41] Sabatti M , Gaudet M, Müller NA, Kersten B, Gaudiano C, Scarascia Mugnozza G, Fladung M, Beritognolo I. Long-term study of a subdioecious *Populus* ×*canescens* family reveals sex lability of females and reproduction behaviour of cosexual plants. Plant Reprod. 2020;33(1):1–17. 31650409 10.1007/s00497-019-00378-5

[jkac071-B42] Samils B , Rönnberg-Wästljung A-C, Stenlid J. QTL mapping of resistance to leaf rust in *Salix*. Tree Genet Genomes. 2011;7(6):1219–1235.

[jkac071-B43] Sanderson BJ , Feng G, Hu N, Carlson CH, Smart LB, Keefover-Ring K, Yin T, Ma T, Liu J, DiFazio SP, et al Sex determination through X–Y heterogamety in *Salix nigra*. Heredity (Edinb). 2021;126(4):630–639. 33510464 10.1038/s41437-020-00397-3 PMC8115673

[jkac071-B44] Slaght JC , Surmach SG, Kisleiko AA. Ecology and conservation of Blakiston’s fish owl in Russia. In: Nakamura F, editor. Biodiversity Conservation Using Umbrella Species. Singapore: Springer; 2018. p. 47–70.

[jkac071-B45] Smart LB , Volk T, Lin J, Kopp RF, Phillips IS, Cameron KD, White EH, Abrahamson LP. Genetic improvement of shrub willow (*Salix* spp.) crops for bioenergy and environmental applications in the United States. Unasylva. 2005;56:51–55.

[jkac071-B46] Stanton BJ , Serapiglia MJ, Smart LB. The domestication and conservation of *Populus* and *Salix* genetic resources. In: Richardson J, Isebrands JG, editors. Poplars and Willows: trees for Society and the Environment. Wallingford (UK): CAB International; 2014. p. 124–199.

[jkac071-B47] Stoof CR , Richards BK, Woodbury PB, Fabio ES, Brumbach AR, Cherney J, Das S, Geohring L, Hornesky J, Mayton H, *et al*. Untapped potential: opportunities and challenges for sustainable bioenergy production from marginal lands in the Northeast USA. Bioenergy Res. 2014;8(2):482–501.

[jkac071-B48] Sulima P , Przyborowski JA, Kuszewska AZałuski D, Jędryczka M, Irzykowski W. Identification of quantitative trait loci conditioning the main biomass yield components and resistance to *Melampsora* spp. in *Salix viminalis* x *Salix schwerinii* hybrids. Int J Mol Sci. 2017;18(3):677. 10.3390/ijms18030677 PMC5372687 28327519

[jkac071-B49] Taylor J , Butler D. R Package ASMap: efficient genetic linkage map construction and diagnosis. J Stat Softw. 2017;79(6):1–29.30220889

[jkac071-B50] Tozawa M , Ueno N, Seiwa K. Compensatory mechanisms for reproductive costs in the dioecious tree *Salix integra*. Botany. 2009;87(3):315–323.

[jkac071-B51] Ueno N , Kanno H, Seiwa K. Sexual differences in shoot and leaf dynamics in the dioecious tree *Salix sachalinensis*. Botany. 2006;84(12):1852–1859.

[jkac071-B52] Ueno N , Seiwa K. Gender-specific shoot structure and functions in relation to habitat conditions in a dioecious tree, *Salix sachalinensis*. J For Res. 2003;8(1):9–16.

[jkac071-B53] Wei S , Yang Y, Yin T. The chromosome-scale assembly of the willow genome provides insight into Salicaceae genome evolution. Hortic Res. 2020;7(1):45. 32257231 10.1038/s41438-020-0268-6 PMC7109076

[jkac071-B54] Yang G , Xu Q, Li W, Ling J, Li X, Yin, T. Sex-related differences in growth, herbivory, and defense of two *Salix* species. Forests. 2020a;11(4):450.

[jkac071-B55] Yang W , Wang D, Li Y, Zhang Z, Tong S, Li M, Zhang X, Zhang L, Ren L, Ma X, et al A general model to explain repeated turnovers of sex determination in the Salicaceae. Mol Biol Evol. 2021;38(3):968–980. 33027519 10.1093/molbev/msaa261 PMC7947767

[jkac071-B56] Yang W , Wang Y, Liu D, Hussain B, Ding Z, Zhao F, Yang X. Interactions between cadmium and zinc in uptake, accumulation and bioavailability for *Salix integra* with respect to phytoremediation. Int J Phytoremediation. 2020b;22(6):628–637. 31899944 10.1080/15226514.2019.1701981

[jkac071-B57] Zhou R , Macaya-Sanz D, Carlson CH, Schmutz J, Jenkins JW, Kudrna D, Sharma A, Sandor L, Shu S, Barry K, et al A willow sex chromosome reveals convergent evolution of complex palindromic repeats. Genome Biol. 2020;21(1):38. 32059685 10.1186/s13059-020-1952-4 PMC7023750

[jkac071-B58] Zhou R , Macaya-Sanz D, Rodgers-Melnick E, Carlson CH, Gouker FE, Evans LM, Schmutz J, Jenkins JW, Yan J, Tuskan GA, et al Characterization of a large sex determination region in *Salix purpurea* L. (Salicaceae). Mol Genet Genomics. 2018;293(6):1437–1452. 30022352 10.1007/s00438-018-1473-y

