# Supplementary material for: Mapping the sex determination region in the Salix F1 hybrid common parent population confirms a ZW system in six diverse species
Source: G3 (Bethesda). 2022 Mar 25;12(6):jkac071. doi: 10.1093/g3journal/jkac071 (PMC9157088; doi:10.1093/g3journal/jkac071)
Supplement: jkac071_Supplementary_Data [file jkac071_supplementary_data.zip › Suppl/Supplemental_Figure_S1_G3-2022-403263.pdf]

13X-358 – 94006 (Female *S. purpurea*)

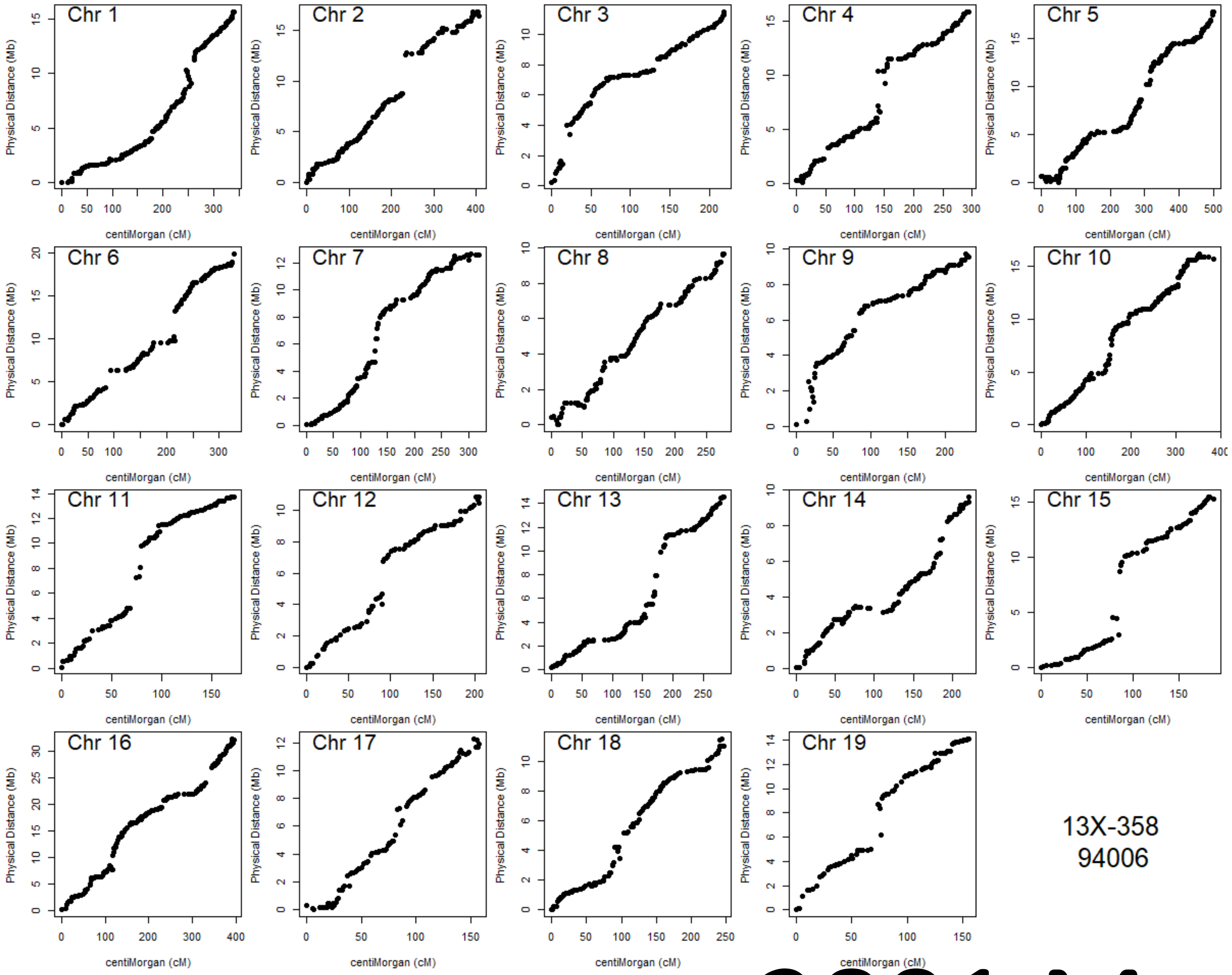

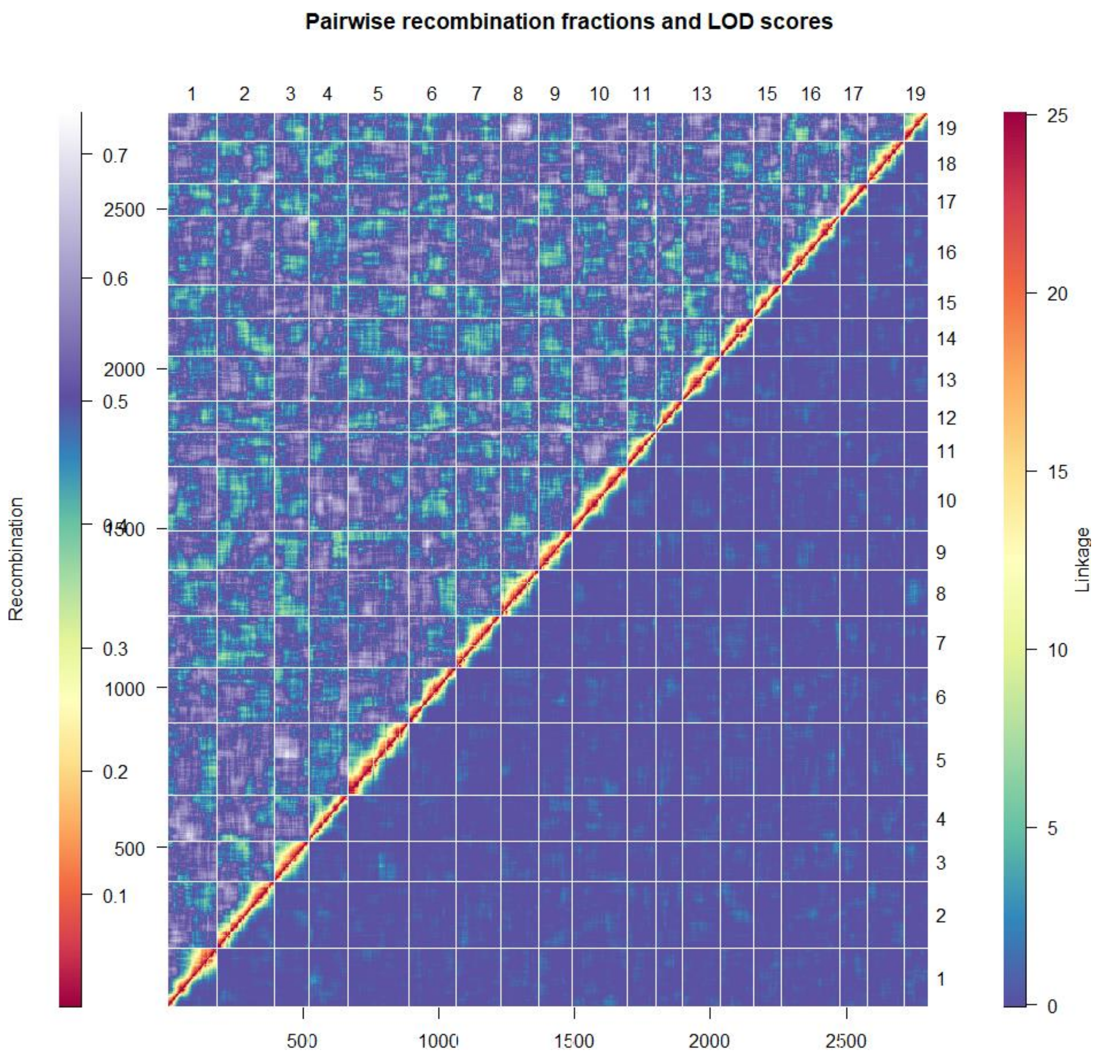

2801 Markers

13X-358 – 04-BN-051 (Male *S. udensis*)

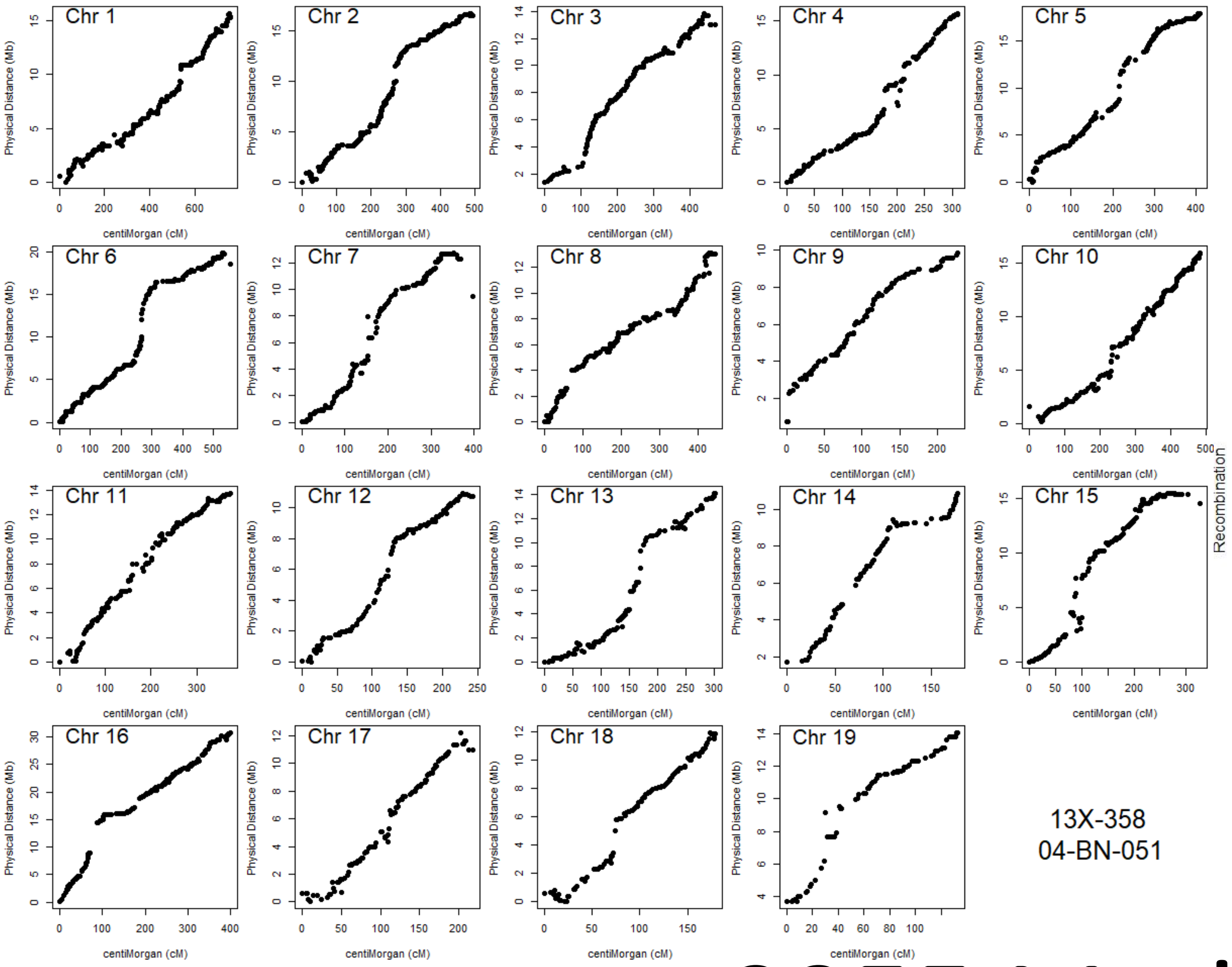

13X-358
04-BN-051

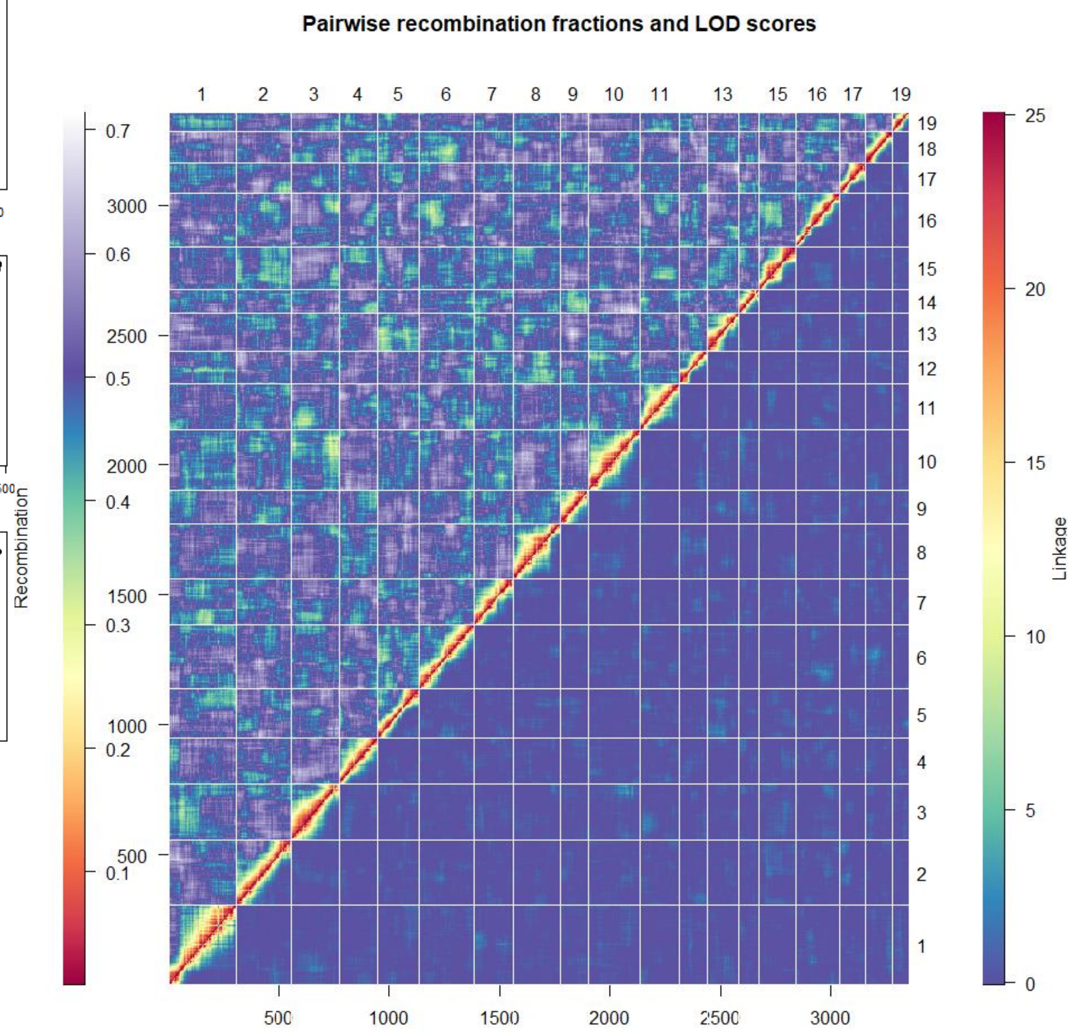

3355 Markers

10X-400 – 94006 (Female *S. purpurea*)

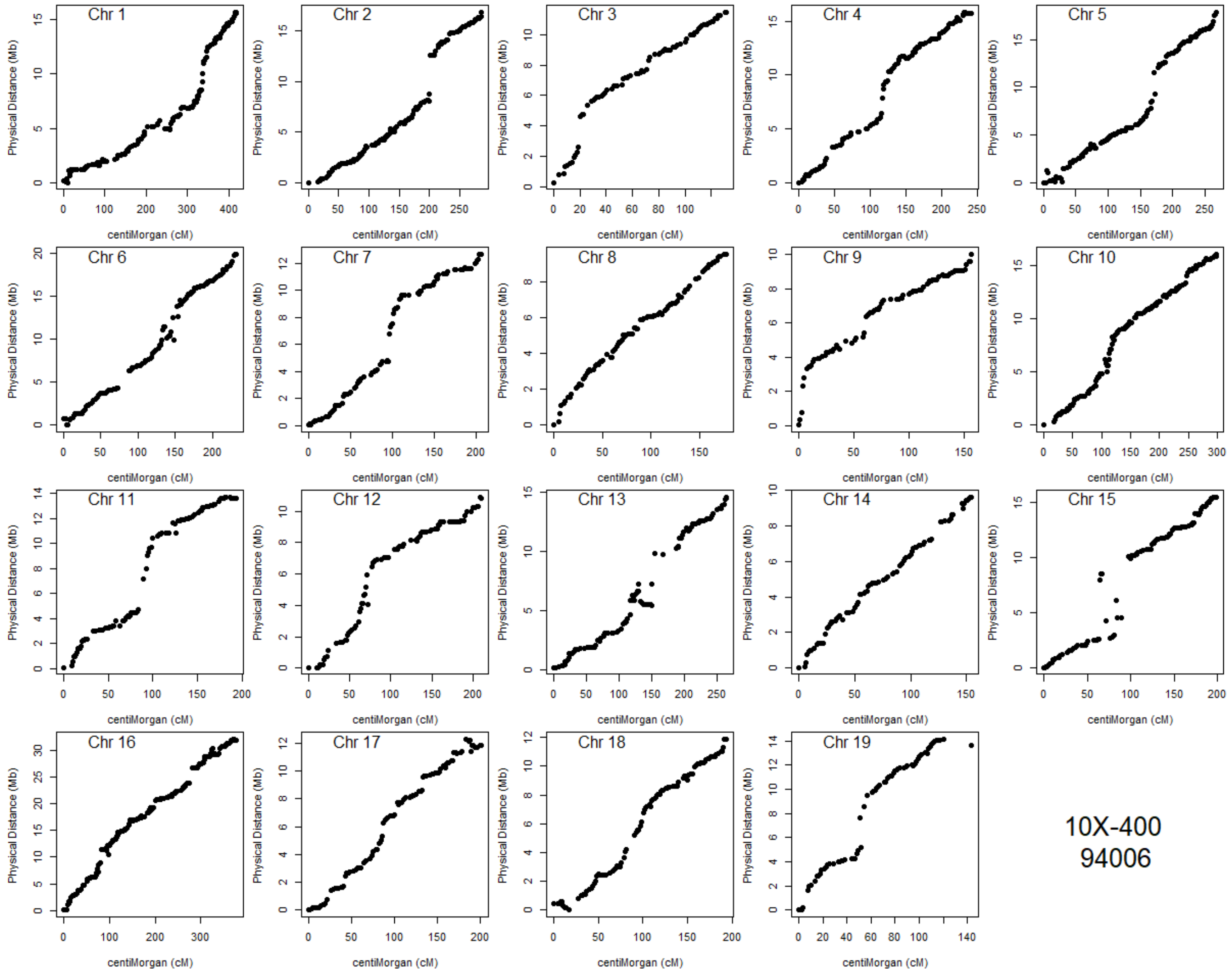

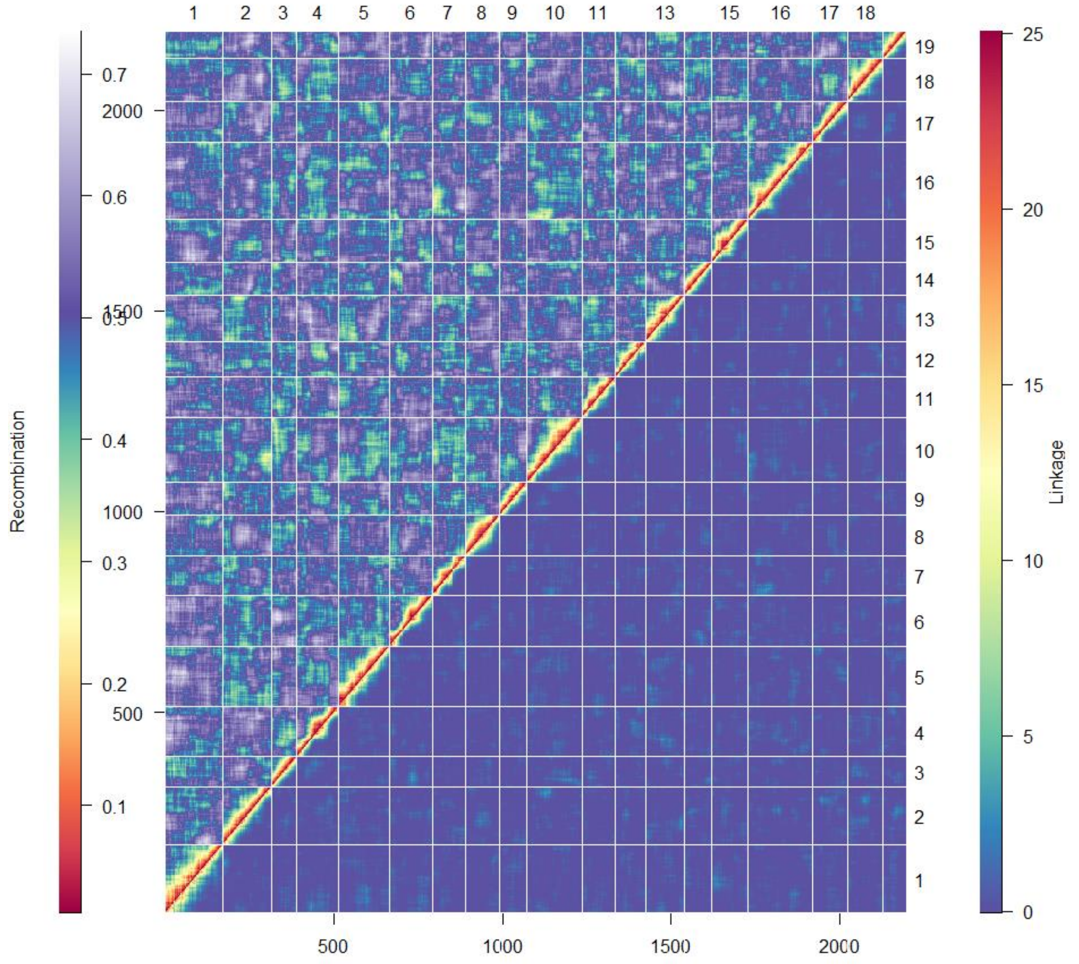

2196 Markers

10X-400 – P63 (Male *S. suchowensis*)

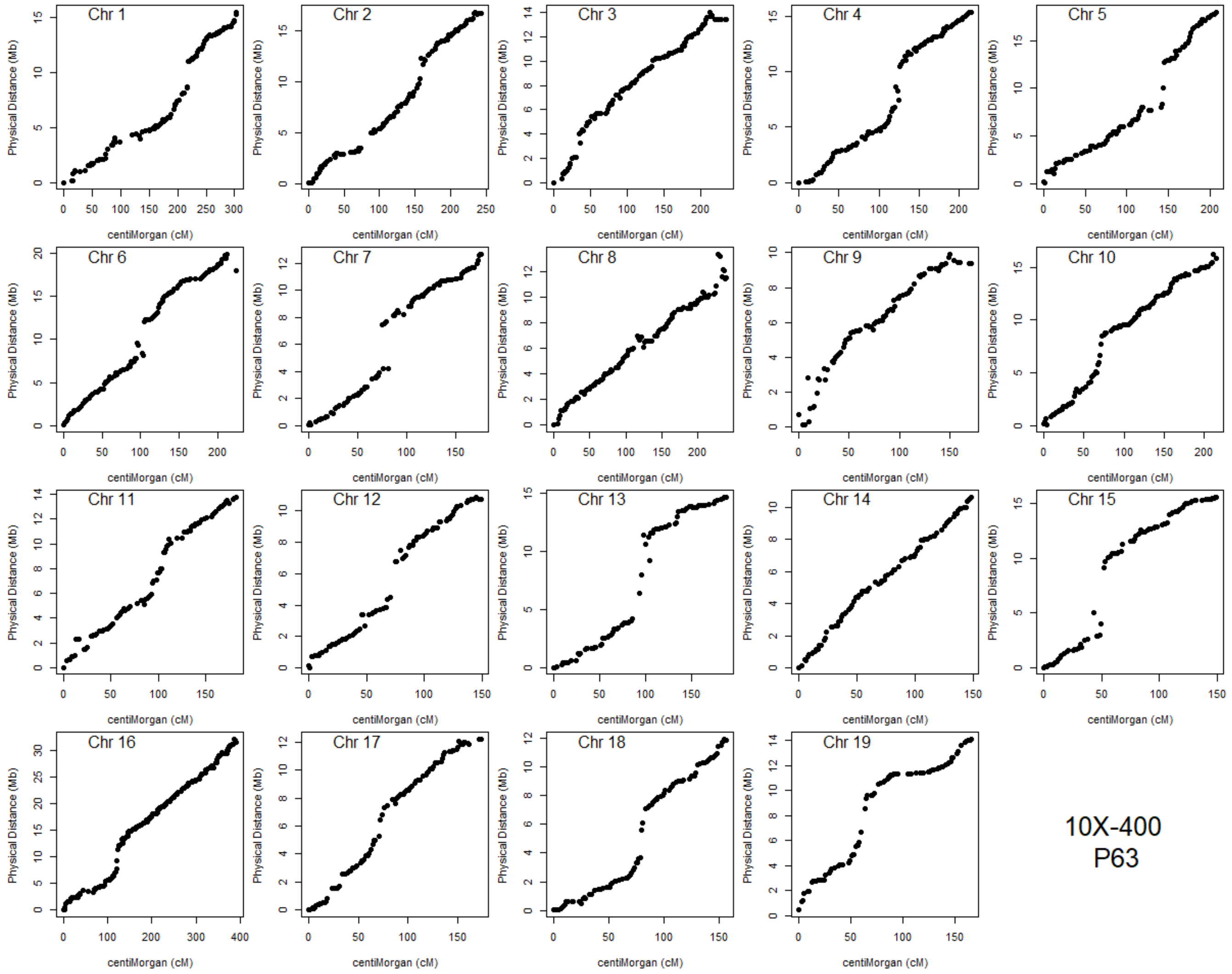

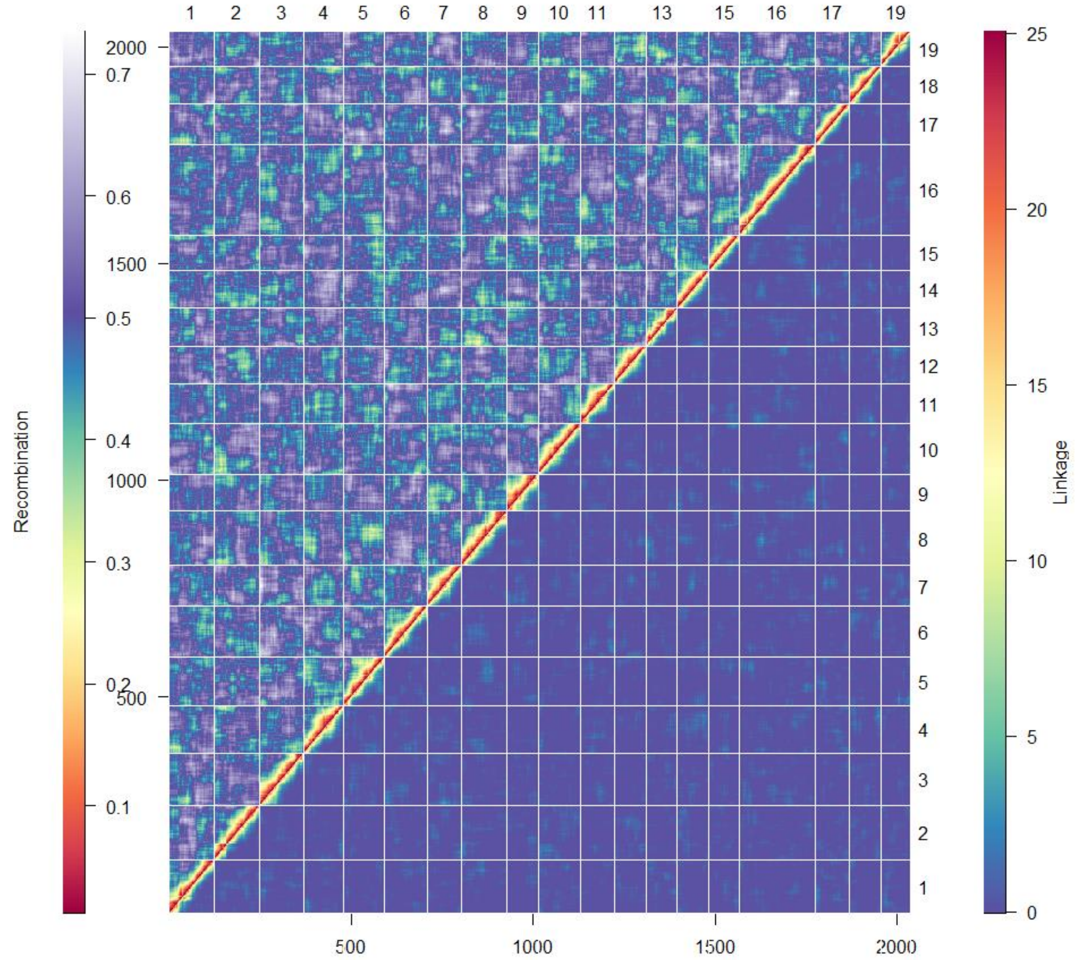

2035 Markers

11X-407 – 94006 (Female *S. purpurea*)

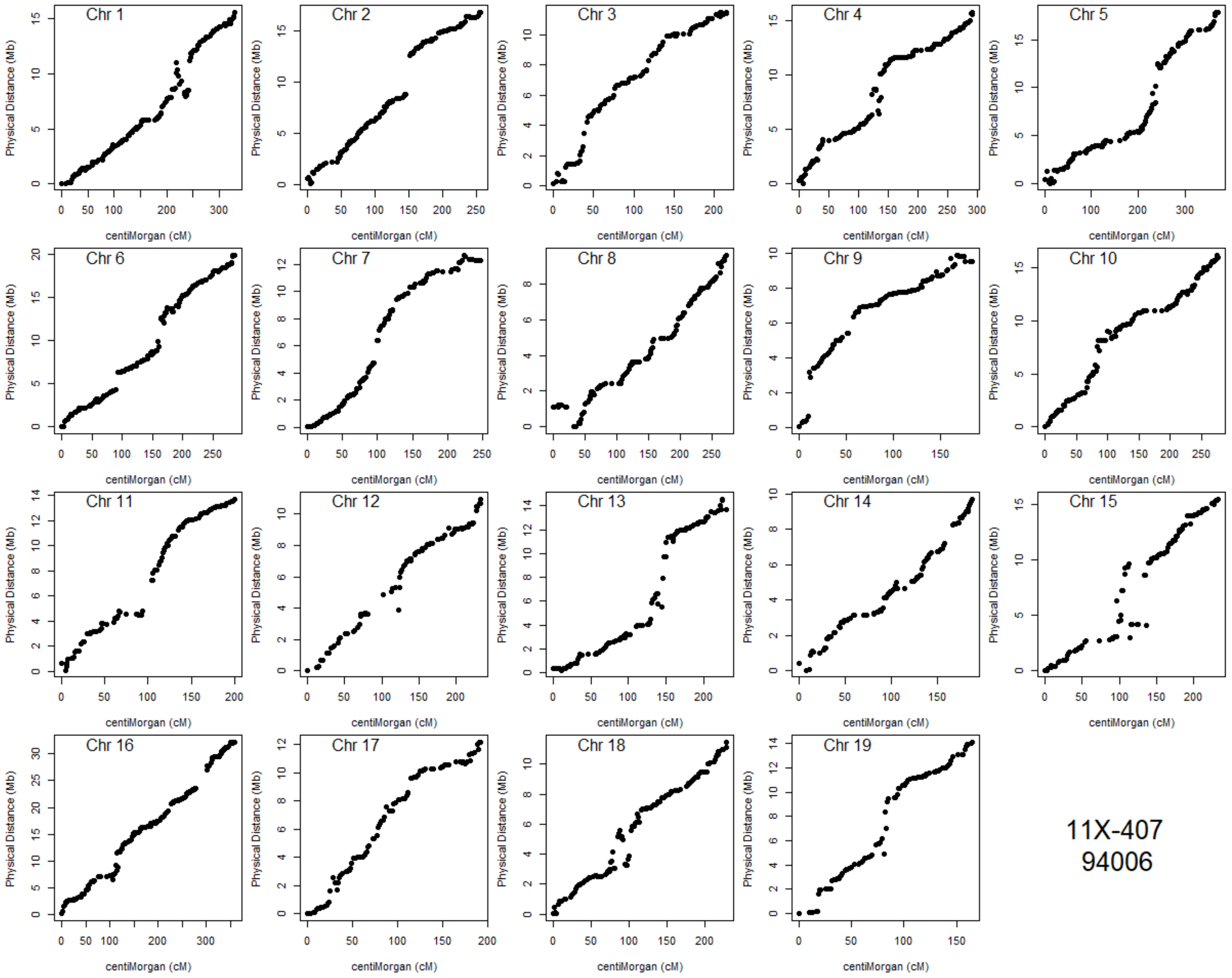

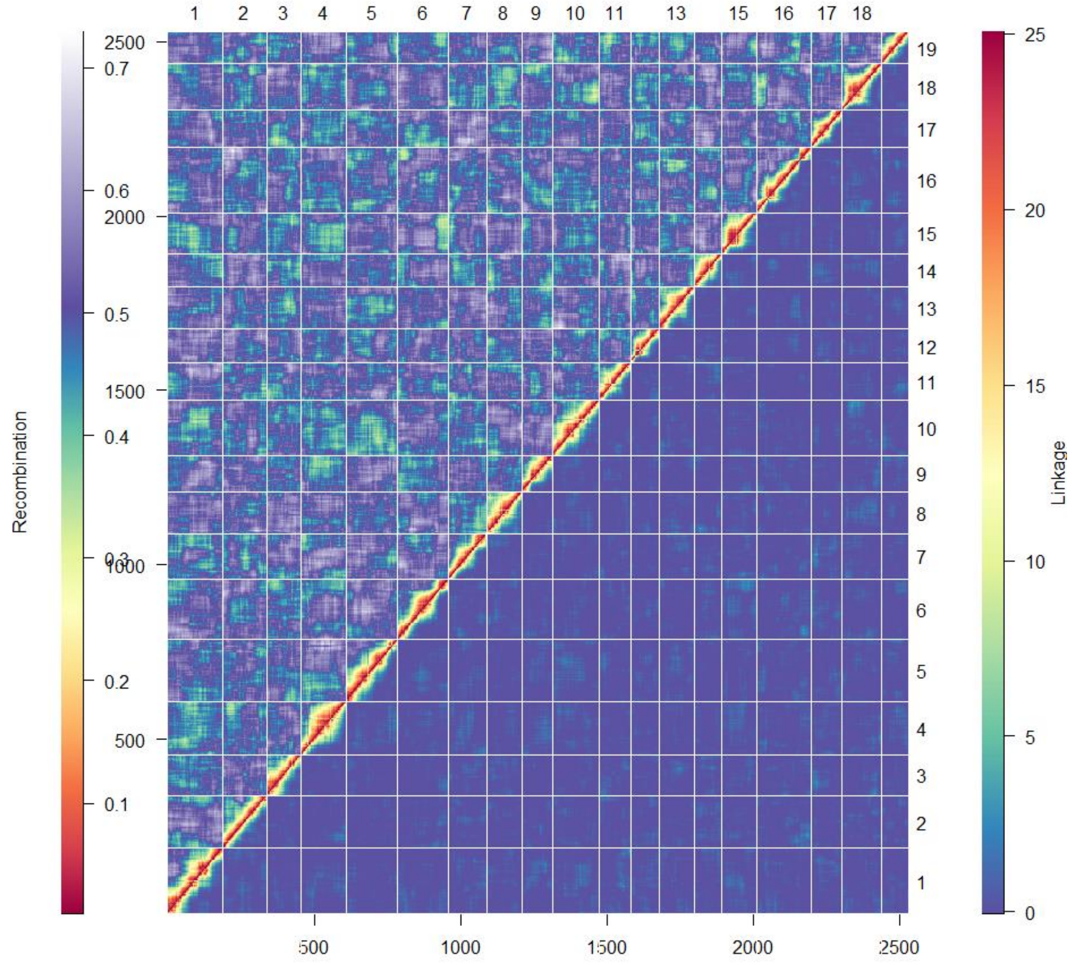

2527 Markers

11X-407 – Jorr (Male *S. viminalis*)

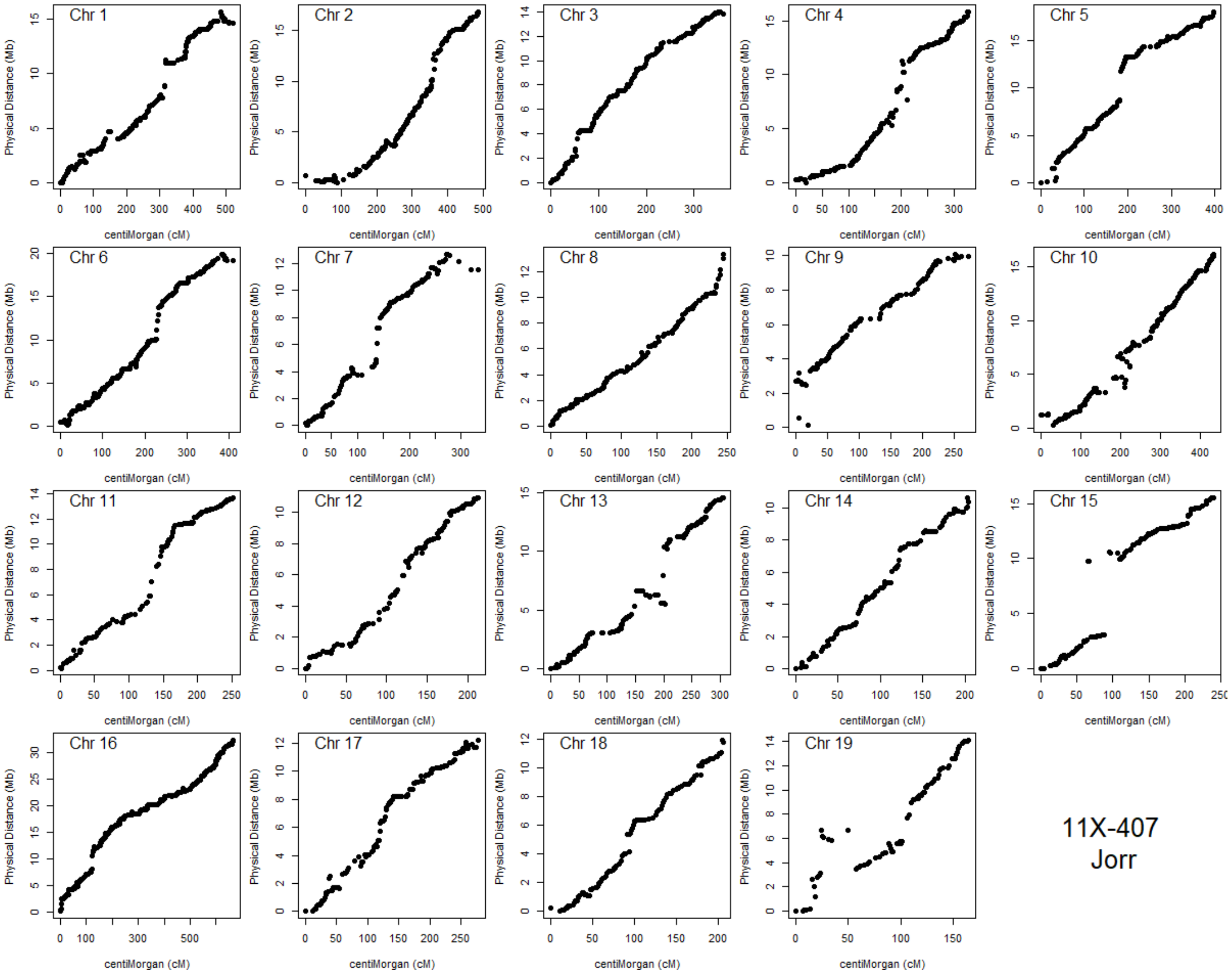

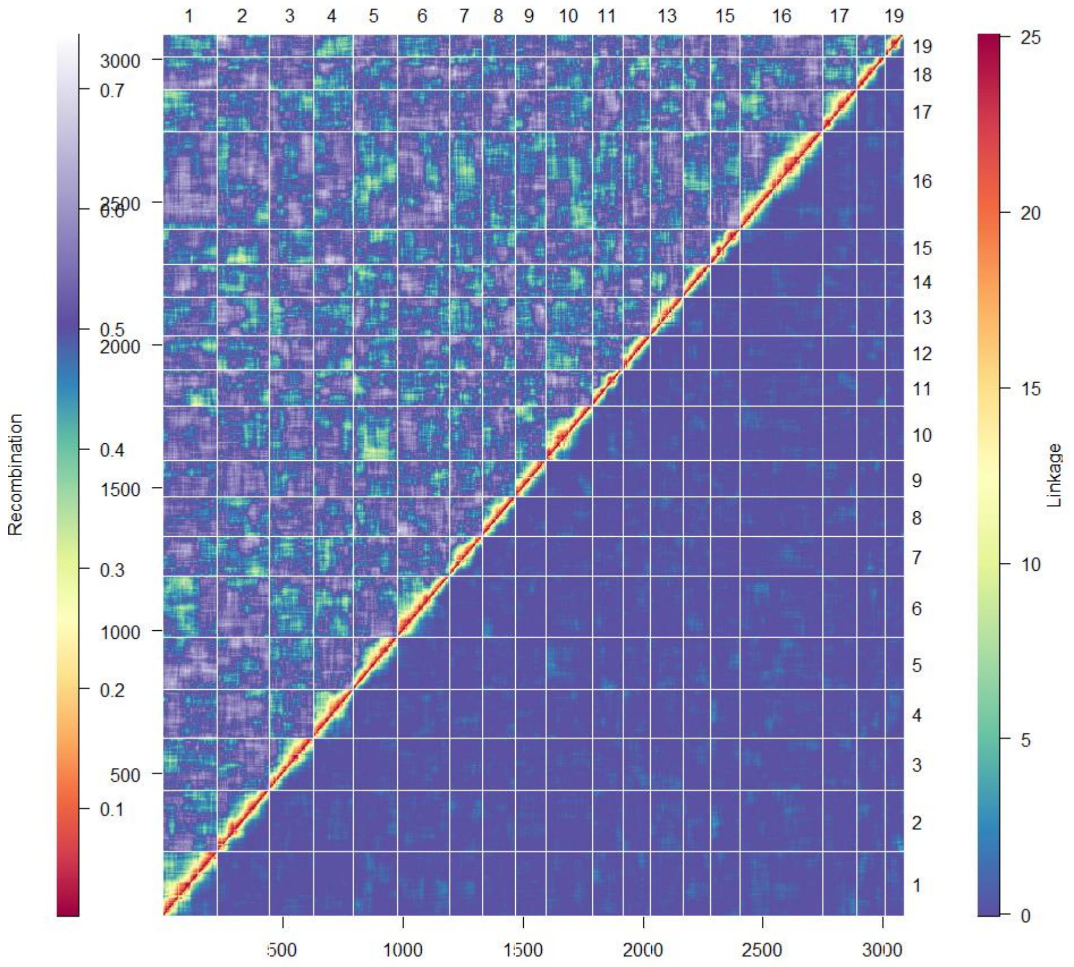

3086 Markers

12X-421 – 07-MBG-5027 (Female *S. viminalis*)

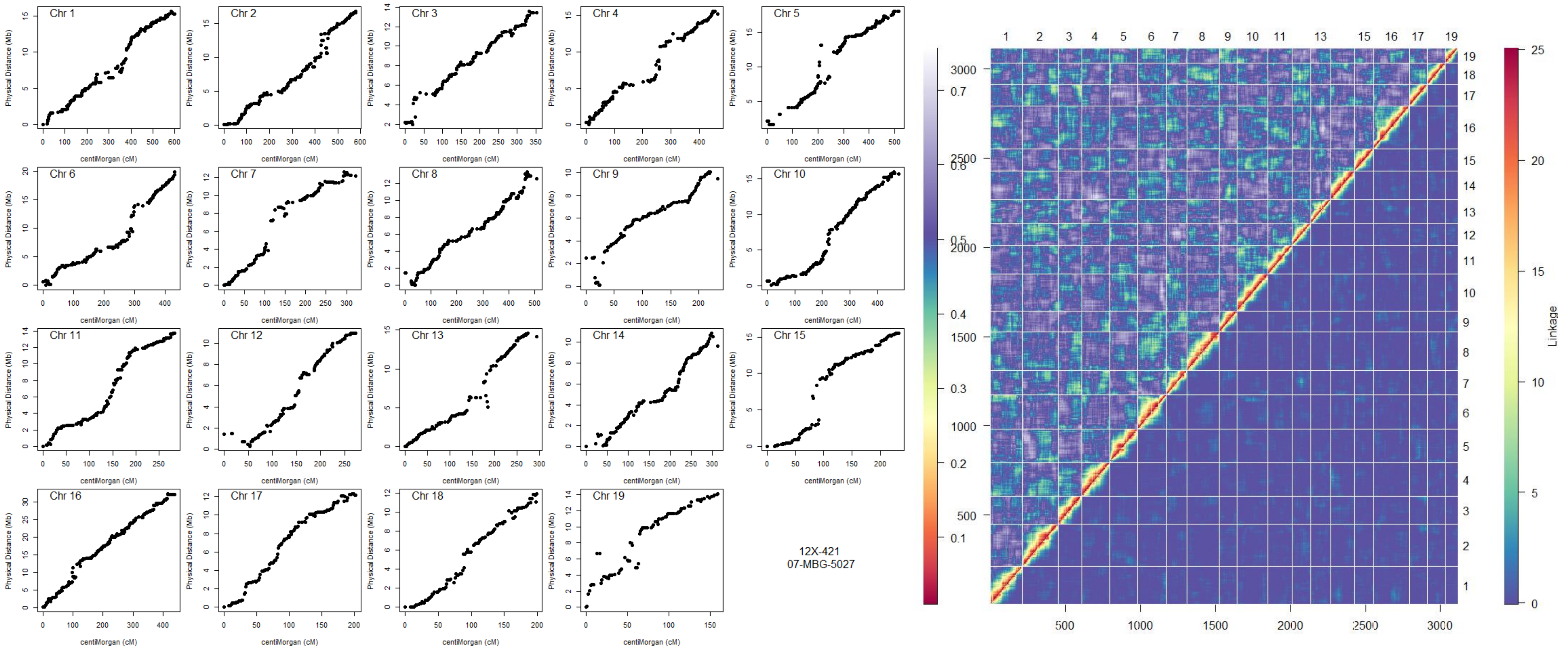

3115 Markers

12X-421 – 94001 (Male *S. purpurea*)

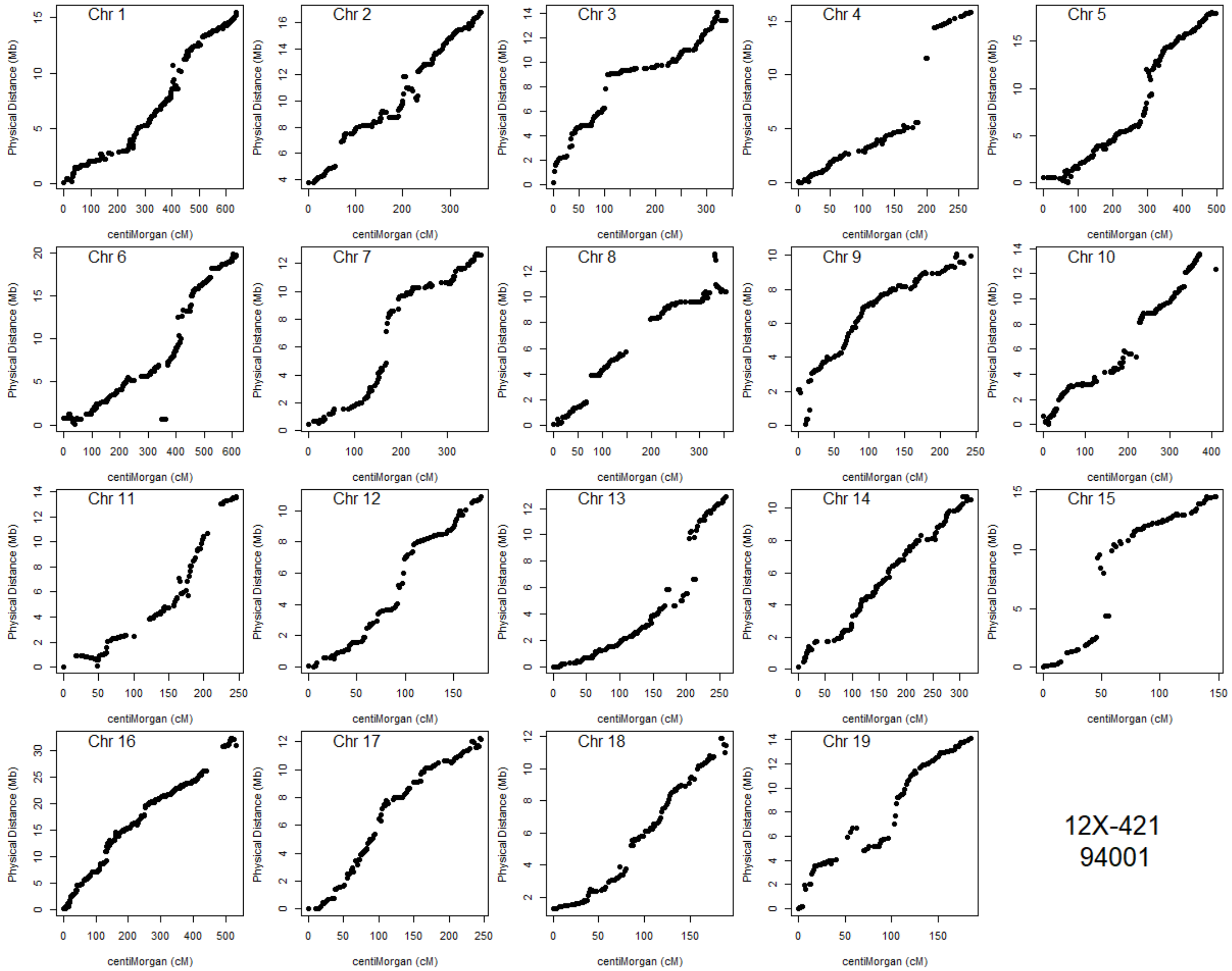

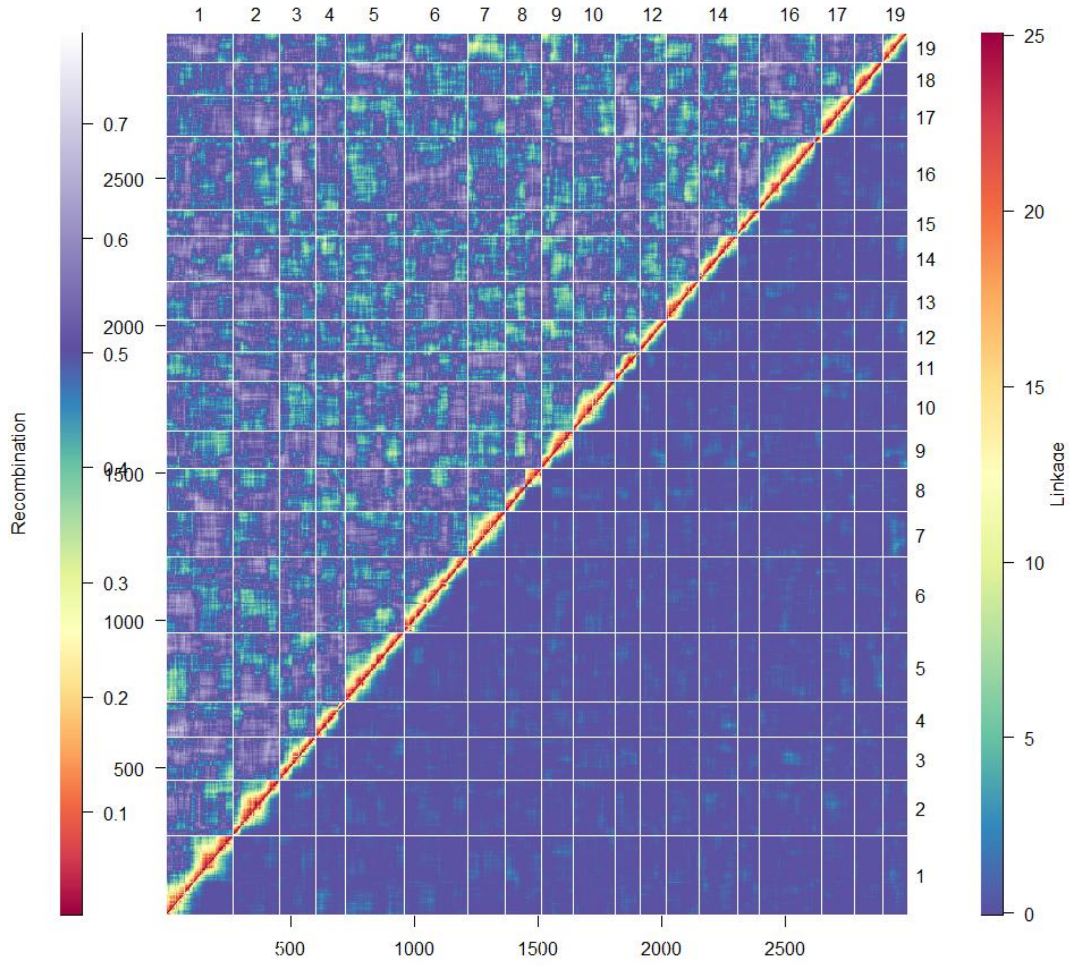

2991 Markers

13X-426 – P336 (Female *S. integra*)

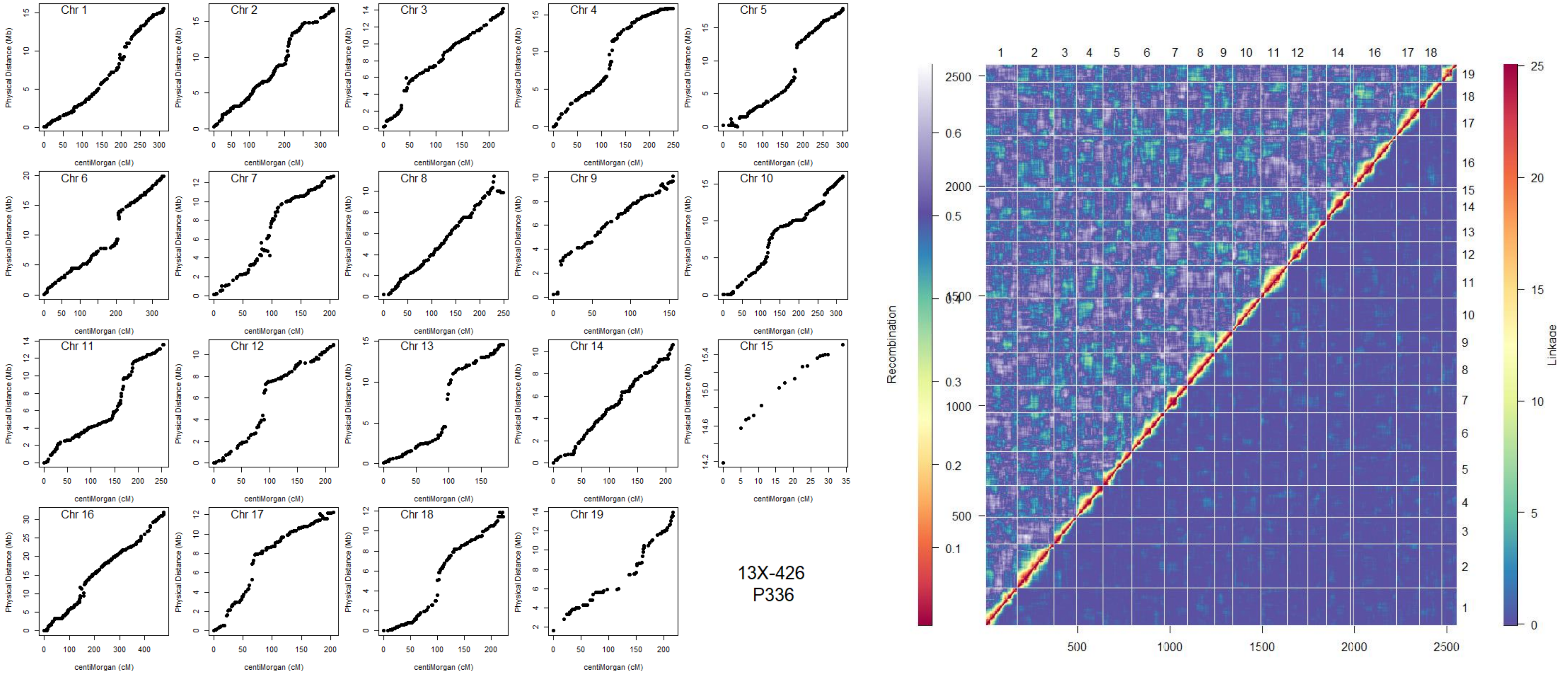

2552 Markers

13X-426 – 94001 (Male *S. purpurea*)

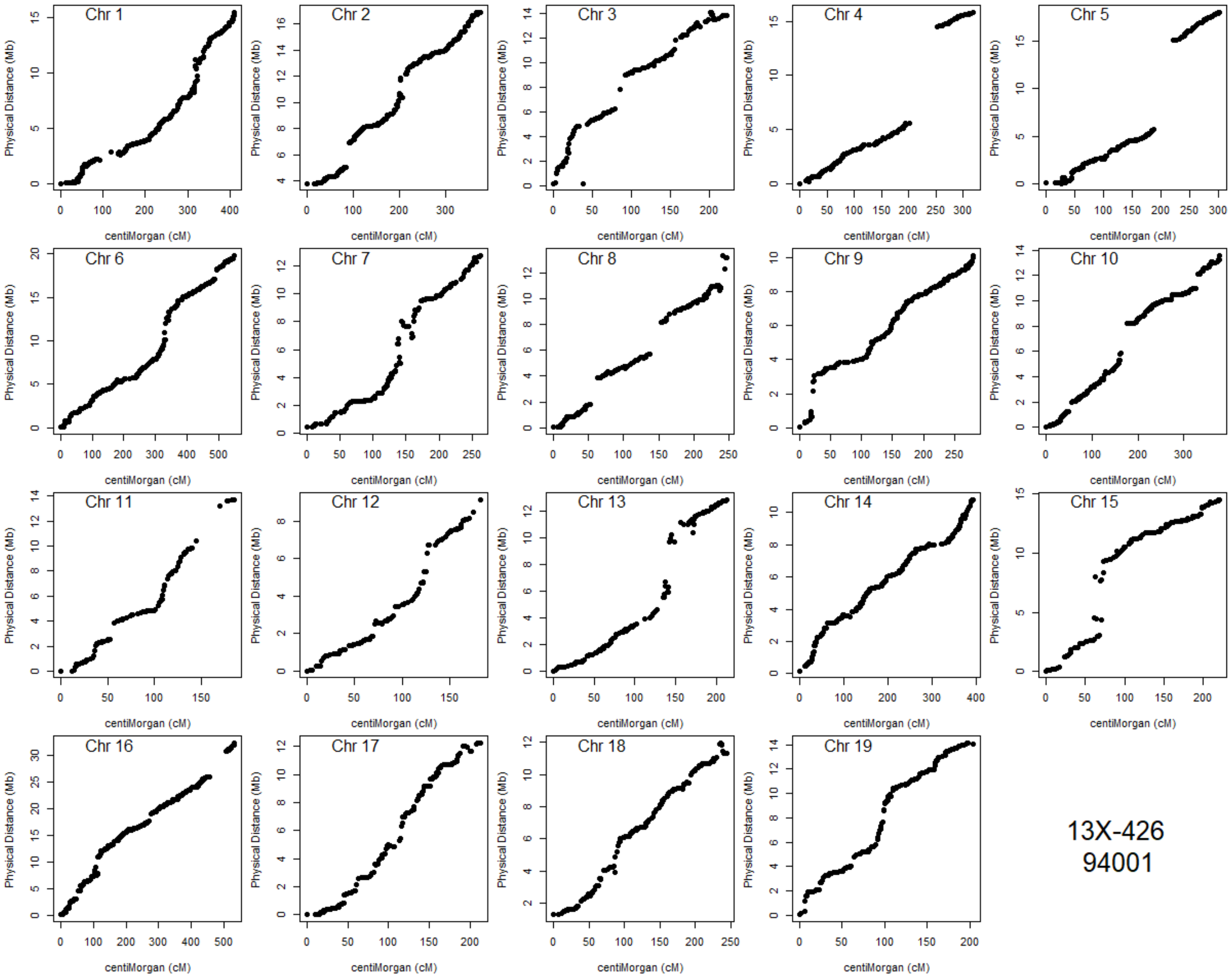

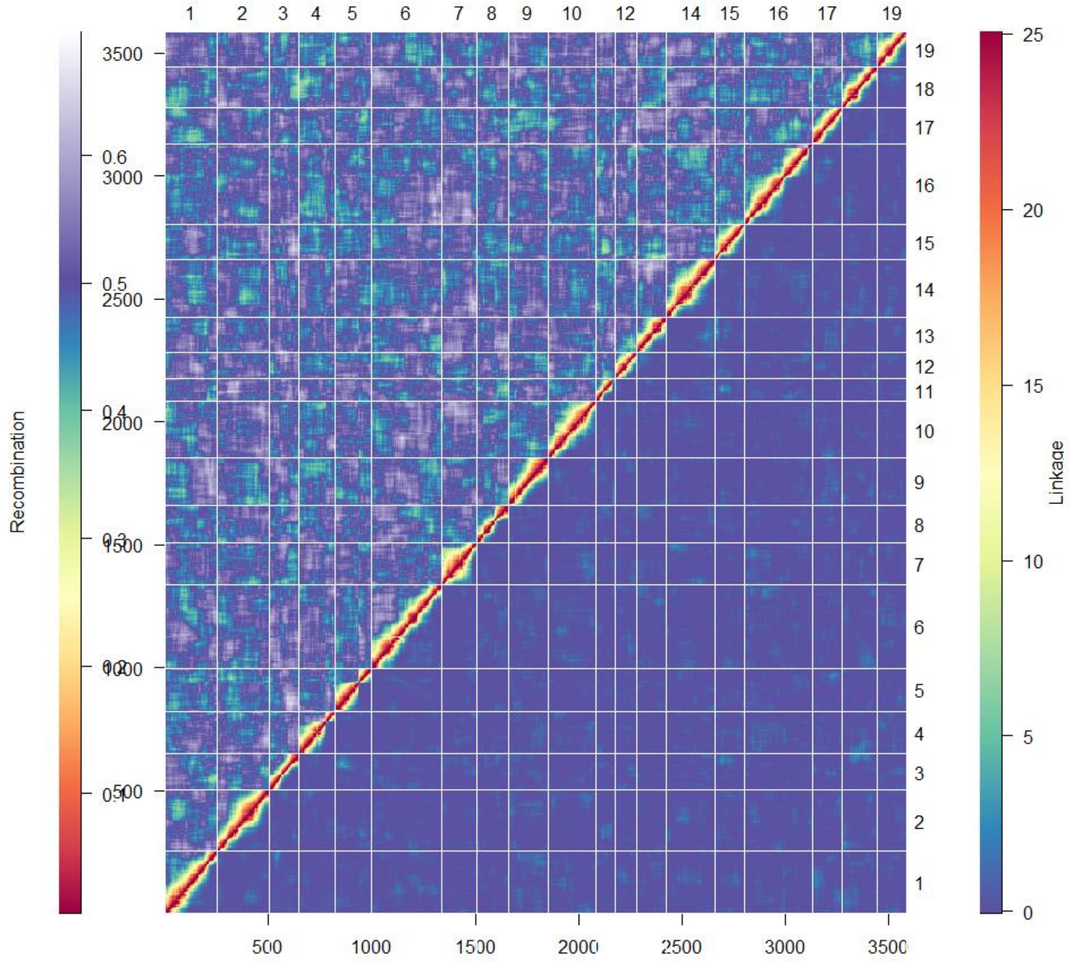

3586 Markers

13X-438 – 94006 (Female *S. purpurea*)

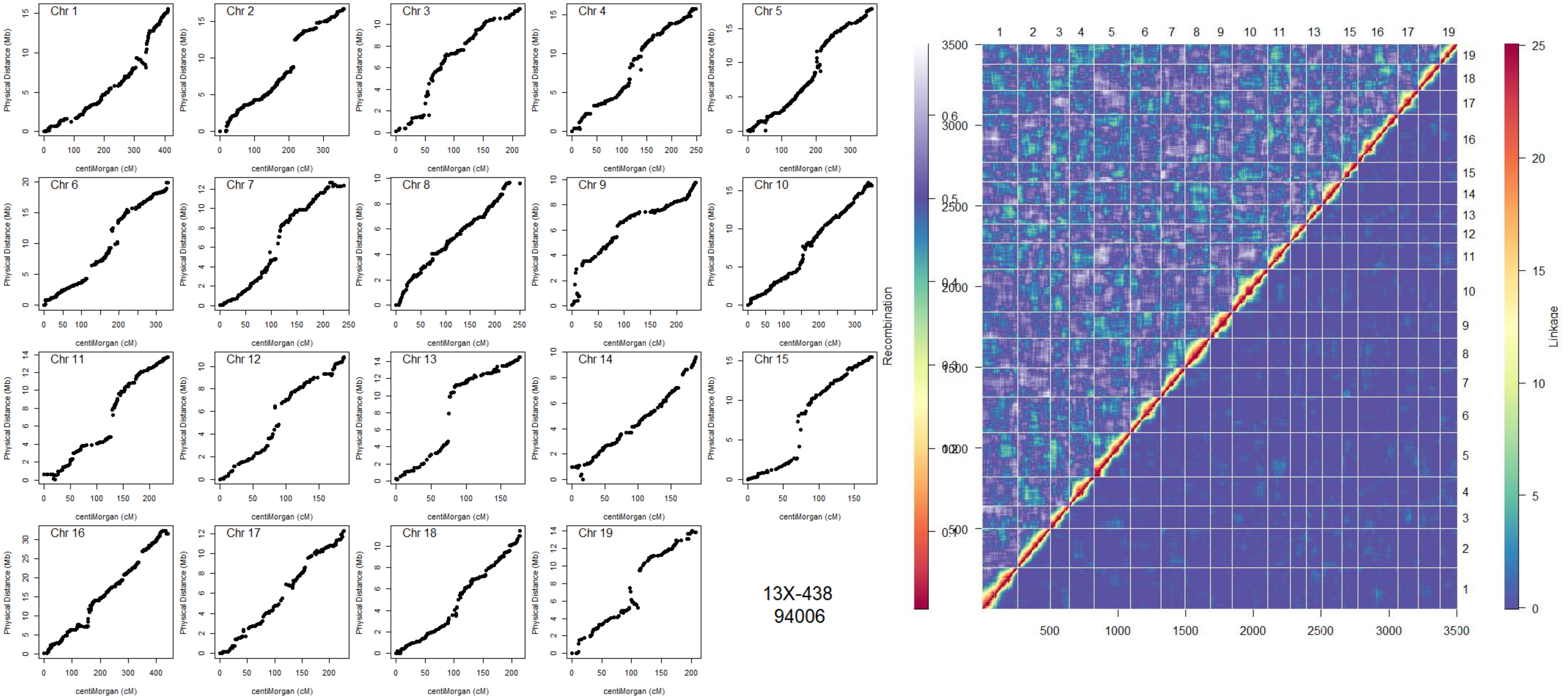

3503 Markers

13X-438 – 04-FF-016 (Male *S. koriyanagi*)

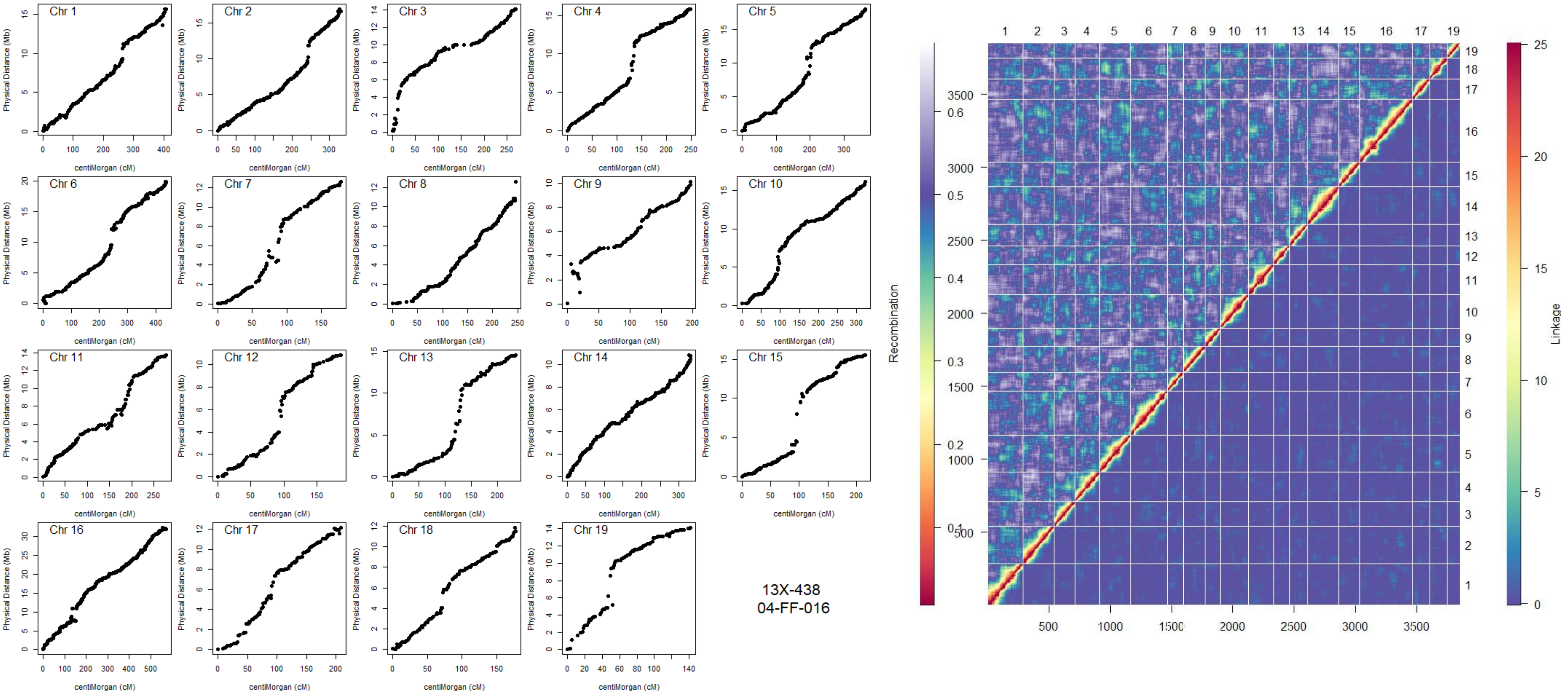

3852 Markers

13X-440 – P295 (Female *S. suchowensis*)

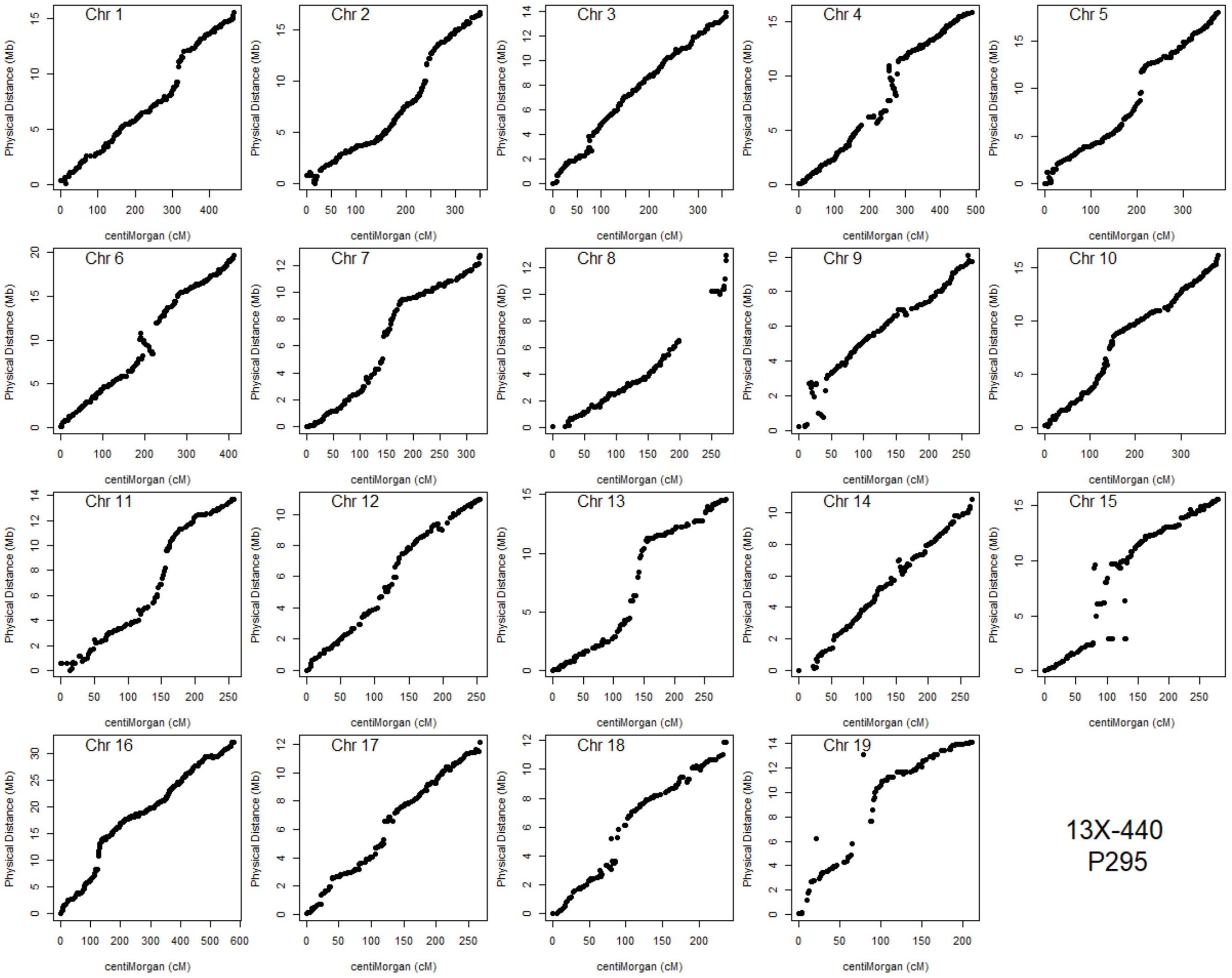

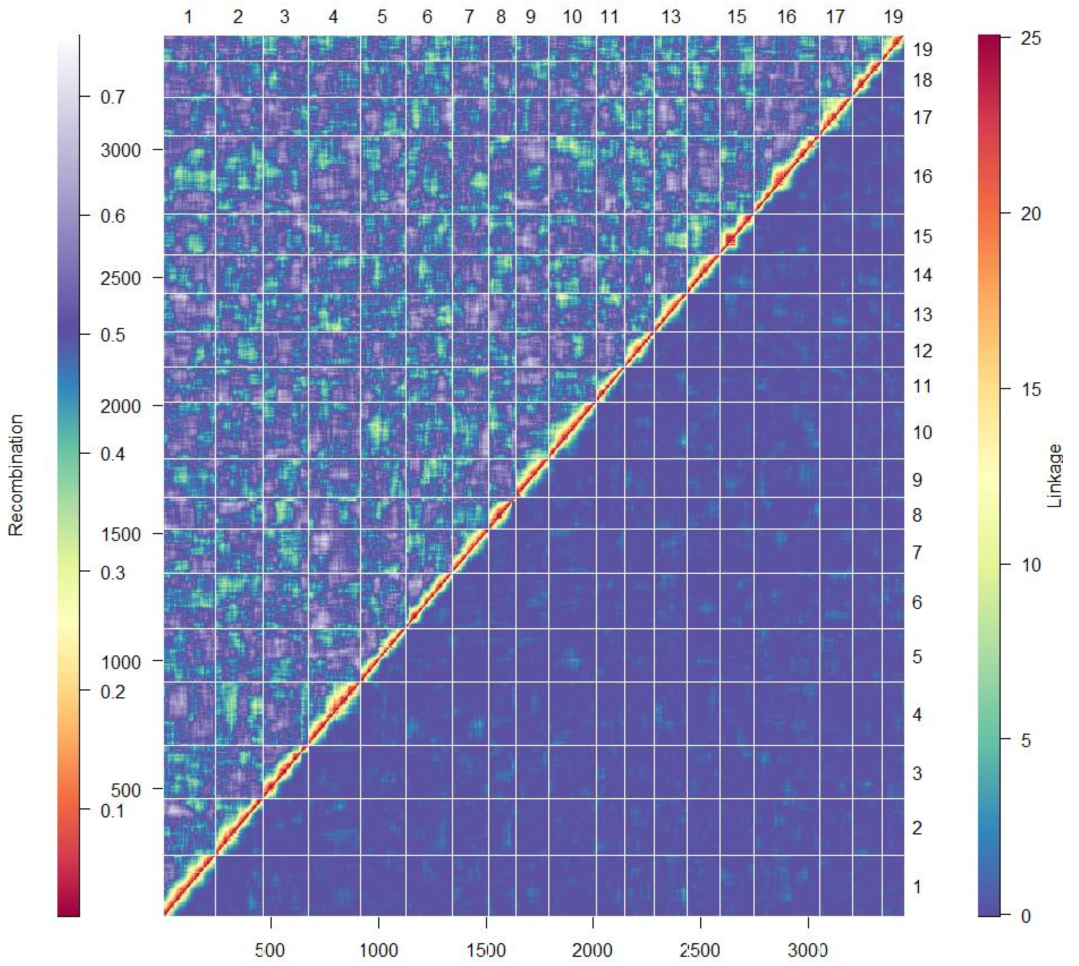

3446 Markers

13X-440 – 94001 (Male *S. purpurea*)

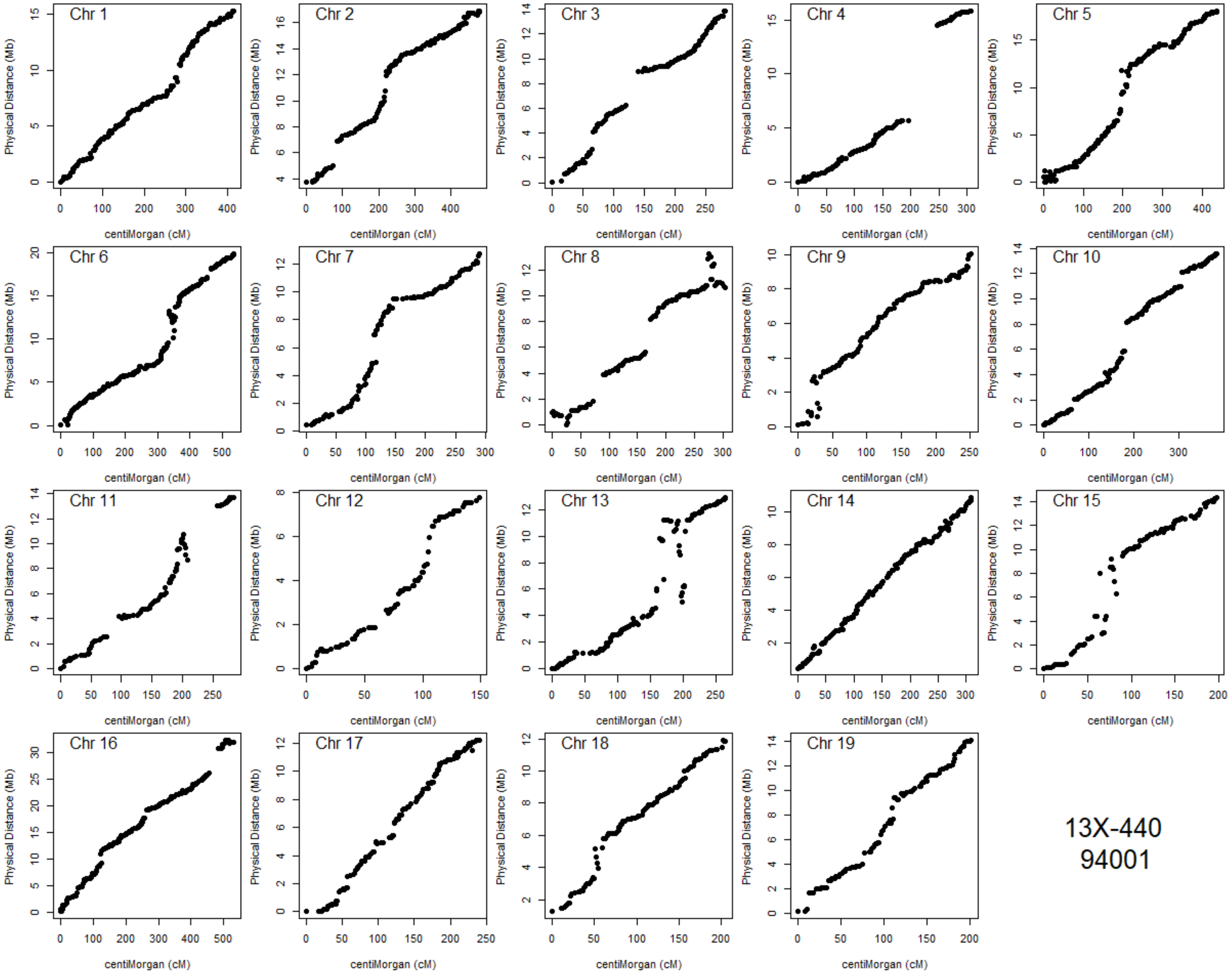

13X-440
94001

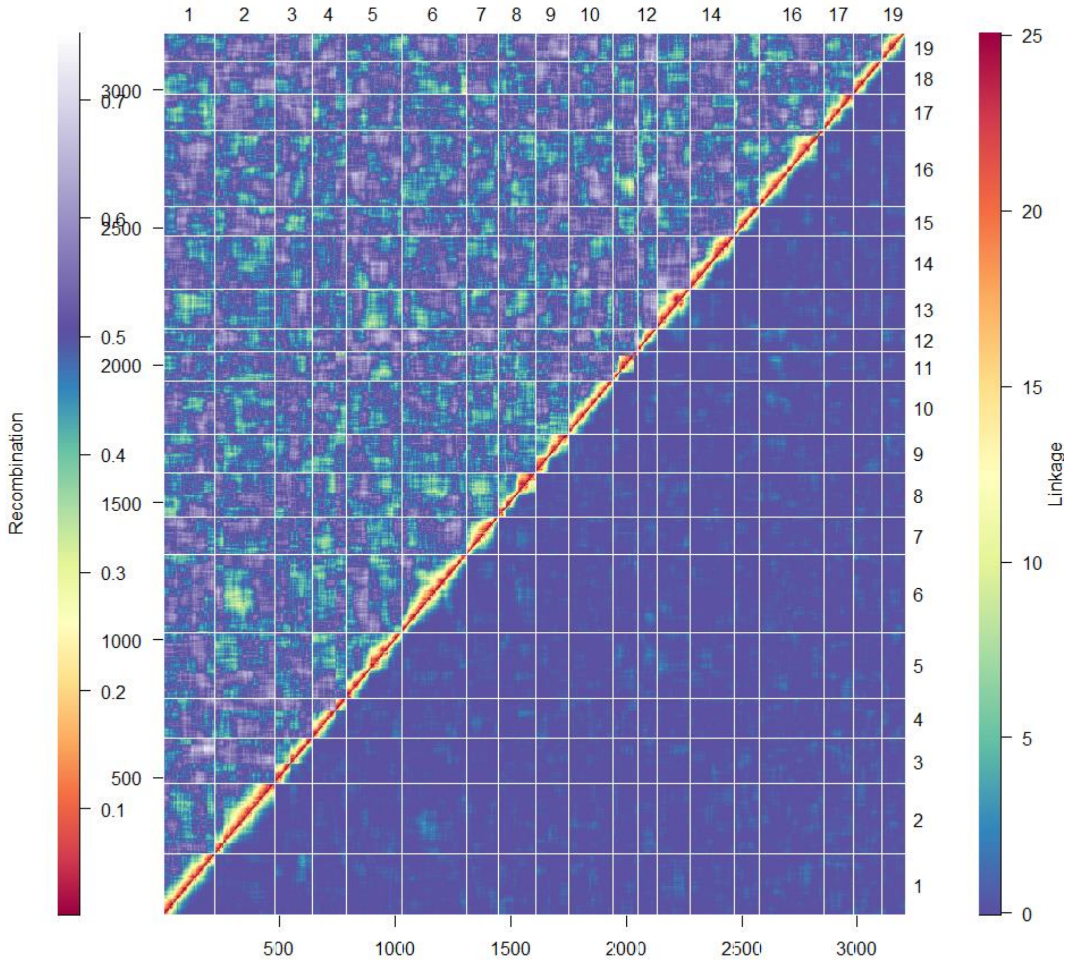

3221 Markers

13X-443 – P294 (Female *S. suchowensis*)

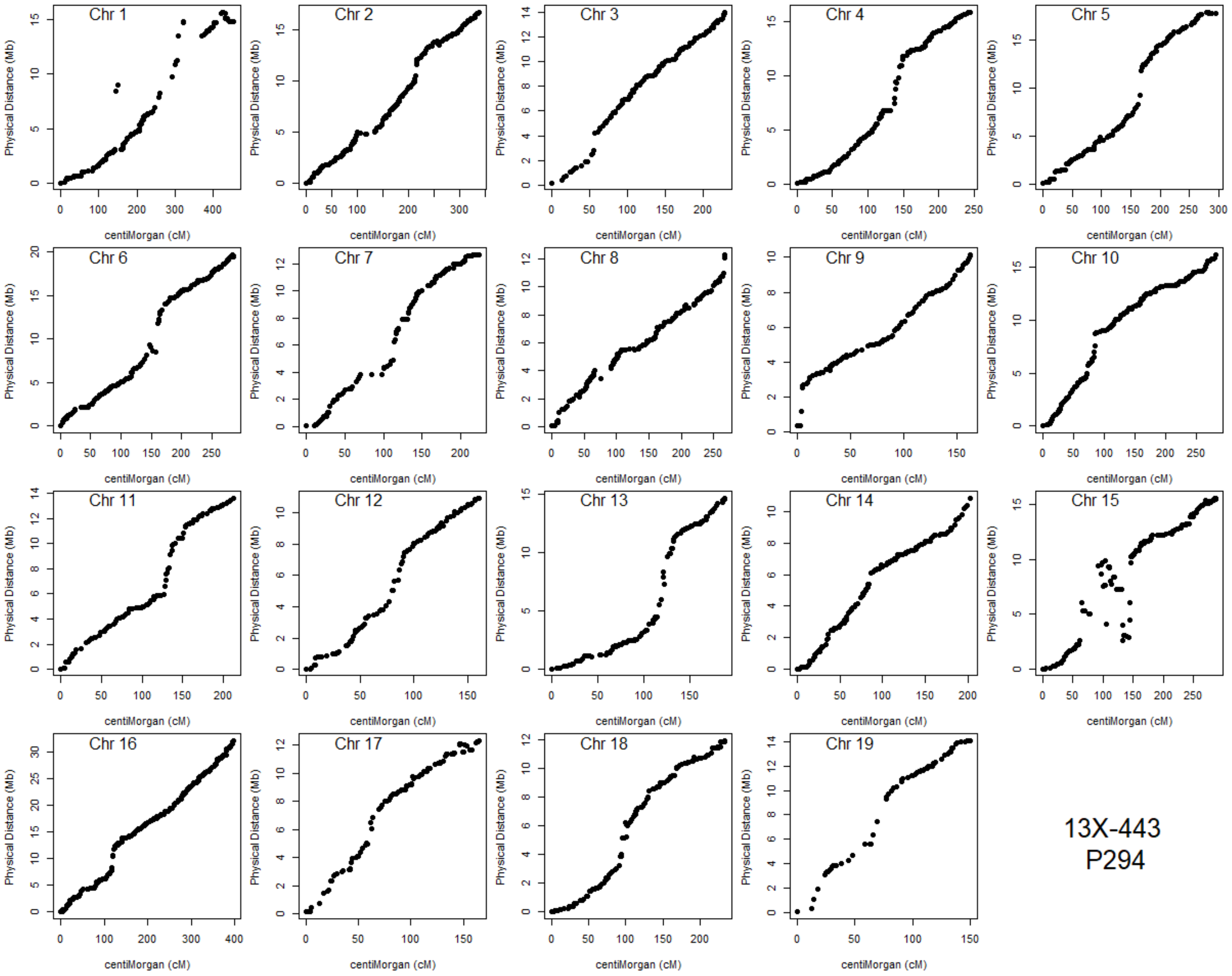

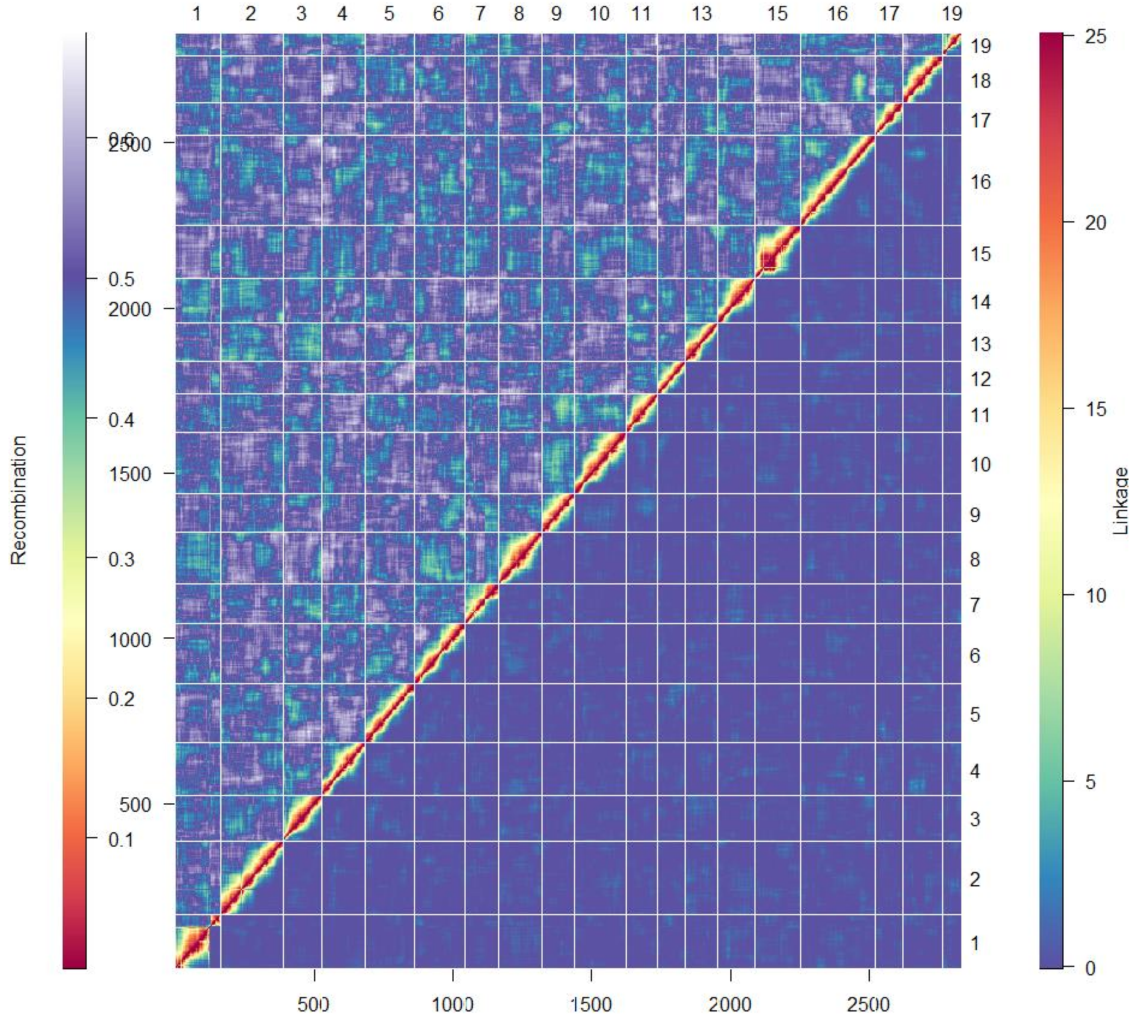

2836 Markers

13X-443 – 94001 (Male *S. purpurea*)

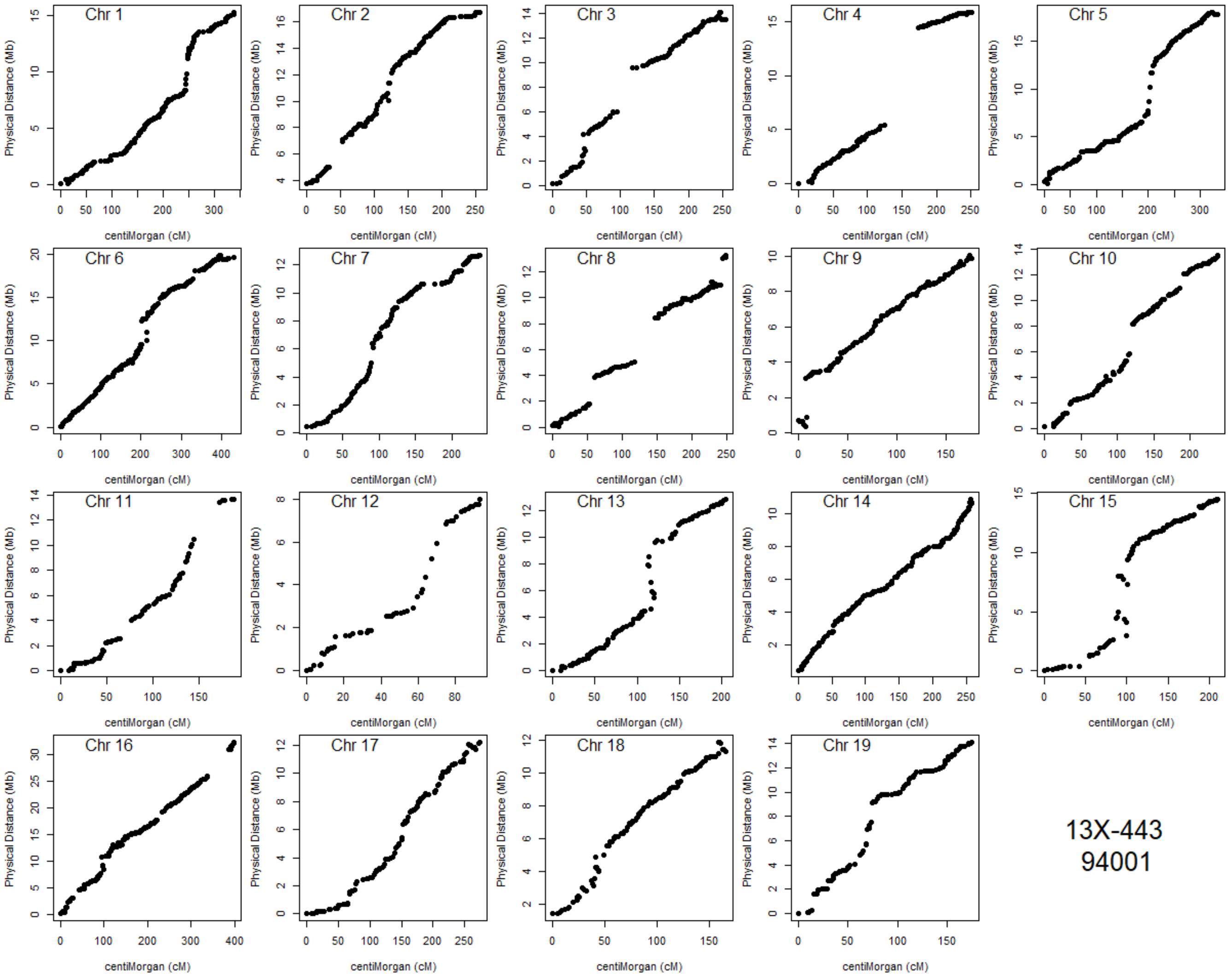

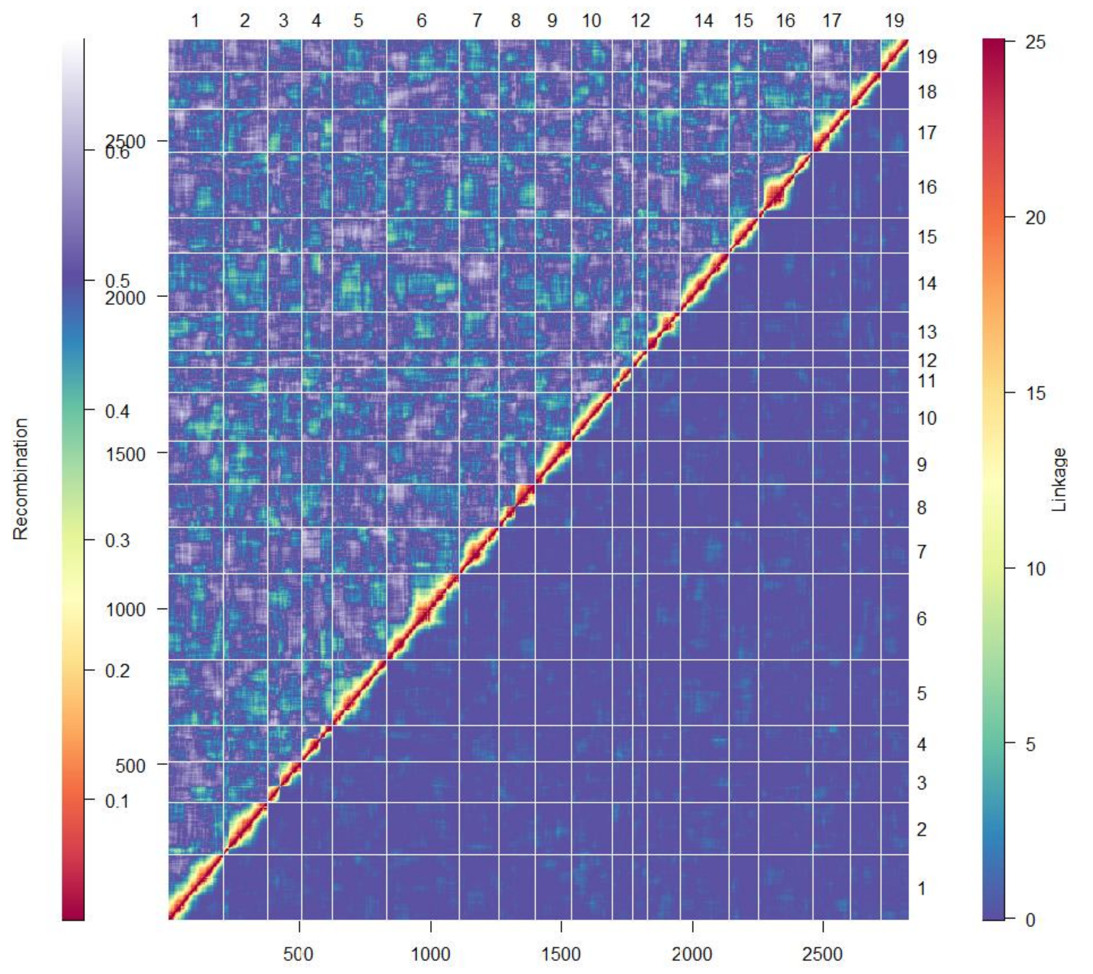

2825 Markers
